# Nontuberculous mycobacterium *M. avium* infection predisposes aged mice to cardiac abnormalities and inflammation

**DOI:** 10.1111/acel.12926

**Published:** 2019-03-04

**Authors:** Colwyn A. Headley, Abigail Gerberick, Sumiran Mehta, Qian Wu, Lianbo Yu, Paolo Fadda, Mahmood Khan, Latha Prabha Ganesan, Joanne Turner, Murugesan V. S. Rajaram

**Affiliations:** ^1^ Department of Microbial Infection and Immunity, College of Medicine The Ohio State University Wexner Medical Center Columbus Ohio; ^2^ Texas Biomedical Research Institute 8715 W. Military Dr. San Antonio TX 78227; ^3^ Department of Microbiology, College of Medicine The Ohio State University Wexner Medical Center Columbus Ohio; ^4^ Department of Biomedical Informatics, College of Medicine The Ohio State University Wexner Medical Center Columbus Ohio; ^5^ Genomics Shared Resource‐Comprehensive Cancer Center, College of Medicine The Ohio State University Wexner Medical Center Columbus Ohio; ^6^ Department Emergency Medicine & Physiology and Cell Biology, College of Medicine The Ohio State University Wexner Medical Center Columbus Ohio; ^7^ Department of Internal Medicine, College of Medicine The Ohio State University Wexner Medical Center Columbus Ohio

**Keywords:** aging, Arrhythmia, ECG, fibrosis, *Mycobacterium avium*, nontuberculous mycobacterium

## Abstract

Biological aging dynamically alters normal immune and cardiac function, favoring the production of pro‐inflammatory cytokines (IL‐1β, IL‐6, and TNF‐α) and increased instances of cardiac distress. Cardiac failure is the primary reason for hospitalization of the elderly (65+ years). The elderly are also increasingly susceptible to developing chronic bacterial infections due to aging associated immune abnormalities. Since bacterial infections compound the rates of cardiac failure in the elderly, and this phenomenon is not entirely understood, the interplay between the immune system and cardiovascular function in the elderly is of great interest. Using *Mycobacterium avium,* an opportunistic pathogen, we investigated the effect of mycobacteria on cardiac function in aged mice. Young (2–3 months) and old (18–20 months) C57BL/6 mice were intranasally infected with *M. avium* strain 104, and we compared the bacterial burden, immune status, cardiac electrical activity, pathology, and function of infected mice against uninfected age‐matched controls. Herein, we show that biological aging may predispose old mice infected with *M. avium* to mycobacterial dissemination into the heart tissue and this leads to cardiac dysfunction. *M. avium* infected old mice had significant dysrhythmia, cardiac hypertrophy, increased recruitment of CD45^+^ leukocytes, cardiac fibrosis, and increased expression of inflammatory genes in isolated heart tissue. This is the first study to report the effect of mycobacteria on cardiac function in an aged model. Our findings are critical to understanding how nontuberculous mycobacterium (NTM) and other mycobacterial infections contribute to cardiac dysfunction in the elderly population.

## 1 INTRODUCTION

Biological aging dynamically transforms the molecular, cellular, and physiological paradigms of homeostasis, often biasing organisms toward dysregulation (Li et al., 2015; Pattabiraman, Palasiewicz, Galvin, & Ucker, 2017; Pomatto & Davies, 2017). In regard to immune function, chronic exposure to noxious environmental stimuli, as well as the intrinsic physiological changes associated with aging, incurs a continual decrease in immune responses that is seemingly coupled with chronic increase in production and circulation of pro‐inflammatory cytokines such as IL‐1β, TNF‐α, and IL‐6. This maladaptation has been termed inflammaging (Franceschi & Campisi, 2014), and this unresolved inflammatory perturbation progressively contributes toward chronic multi‐system dysfunctions (Parkinson disease, Alzheimer, cardiovascular diseases [CVD], arthritis, and diabetes) that impact normal neurological, pulmonary, vascular, immunological metabolic and cardiac status (Xia et al., 2016).

Cardiac failure (CF) is the most common cause of hospitalization in patients 65 years or older (Blecker, Paul, Taksler, Ogedegbe, & Katz, 2013). Elderly CF is associated with increased prevalence of coronary disease, hypertension, and diabetes (Biernacka & Frangogiannis, 2011). Additionally, the rate of CF in the elderly is critically increased by the presence of bacterial infections (Court, Kumar, Parrillo, & Kumar, 2002; Drosatos et al., 2015; Parrillo et al., 1990; Rudiger & Singer, 2007). Bacterial sepsis induced multi‐organ dysfunction syndrome (MODS), which encompasses CF, carries a 70% mortality rate in hospitalized patients (Court et al., 2002; Parrillo et al., 1990; Rudiger & Singer, 2007).

We have previously shown that pulmonary infections with the bacterium *Franscisella tularensis subspecies novicida* (*Ft.n*) induced cardiac damage. More specifically, intranasal infection of mice with *Ft.n* resulted in altered cardiac electrophysiology (increased heart rate, QRS duration, and PR intervals), significant formation of cardiac micro‐lesions, cardiac fibrosis, as well as immune cell infiltration, myocarditis, and bacterial colonization of cardiac tissue (Makara et al., 2016). Studies by other groups investigating bacterial infections associated with sepsis and cardiac failure (Bergounioux et al., 2016; Brown et al., 2014) have paralleled our findings. Additionally, bacterial products can activate toll‐like receptor‐mediated inflammatory cytokines that contribute to cardiomyocyte contractile dysfunction and subsequent cardiac damage (Boyd, Mathur, Wang, Bateman, & Walley, 2006; Fillon et al., 2006; Rolli et al., 2010). In short, there is growing consensus that the dissemination of bacteria into cardiac tissue may be a pivotal step in the onset of cardiac failure in sepsis patients. Dissecting the underlying interactions among bacterial infections, their immuno‐pathological consequences and cardiac function in old age are necessary to develop effective therapeutic strategies.

In this study, we examined cardiac function of young and old mice during nontuberculous mycobacteria (NTM) infection. NTM are usually opportunistic pathogens and are ubiquitously found in nature (Bermudez, Wagner, & Sosnowska, 2000; Whiley, Keegan, Giglio, & Bentham, 2012). Individuals with chronic lung infections such as patients with HIV, COPD, cystic fibrosis, and/or medications that dampen proper immune response have increased susceptibility to contract NTM infections. Nevertheless, age is also a major prognostic factor for contraction of NTM disease and recent clinical data reflect increased incidences in elderly immunocompetent individuals (Prevots & Marras, 2015; Stout, Koh, & Yew, 2016).

The NTM species of the *Mycobacterium avium *complex (MAC*), M. kansasii* and *M. abscessus* have been implicated in pulmonary human disease (McShane & Glassroth, 2015), with MAC being a principal culprit of clinically diagnosed pulmonary NTM infections (Mirsaeidi, Farshidpour, Ebrahimi, Aliberti, & Falkinham, 2014; Prevots & Marras, 2015; Schluger, 2007). Despite *M. avium* pathogenesis not being entirely delineated in humans, it is arguably one of the most characterized and studied NTM (Appelberg, 1994, 2006; Appelberg et al., 1994; Bermudez et al., 2000; Field, Fisher, & Cowie, 2004; Johnson & Odell, 2014; Morimoto et al., 2016; Stout et al., 2016). Typical contraction of *M. avium* stems from the inhalation or ingestion of aerosolized mycobacteria or mycobacterial droplets (Appelberg, 1994, 2006; Appelberg et al., 1994; Bermudez et al., 2000). If *M. avium* successfully bypasses host defense mechanisms in the pulmonary cavity or intestinal tract, mycobacteria can persist by surviving in tissue resident cells such as macrophages and epithelial cells (Reddy, 1998; Sangari, Goodman, & Bermudez, 2000). In fact, the innate and adaptive immune responses to *M. avium* share some semblance to that of pathogenic *M. tuberculosis *in regard to the involvement of macrophages and CD4^+^ T cells that may contribute to a chronic diseased state (Appelberg, 2006; Bermudez et al., 2000). Once internalized by tissue resident macrophages, *M. avium *can inhibit phagosome acidification, is seemingly impervious to the antimicrobial oxidative burst (ROS) and nitric oxide (NO), can persist and multiply within the phagocytic vacuole of resting macrophages, and can induce the production of immuno‐suppressive cytokines such as IL‐10 and TGF‐β (Appelberg, 1994, 2006; Appelberg et al., 1994; Bermudez et al., 2000).

Herein, we have uncovered that *M. avium* infection causes premature atrial contraction and cardiac dysrhythmia in old mice. Intranasally infected old mice suffered from mycobacterial dissemination in the superior pericardium, increased infiltration of immune cells and cardiac fibrosis in the heart. We additionally show an increased expression of inflammatory genes and genes related to cardiac fibrosis in the heart of *M. avium *infected old mice. The findings from this NTM infection model are critical to further our comprehension of cardiac dysfunction in the elderly.

## 2 RESULTS

### 2.1 *M. avium* disseminates into the cardiac tissue of old mice

**Figure 1:**
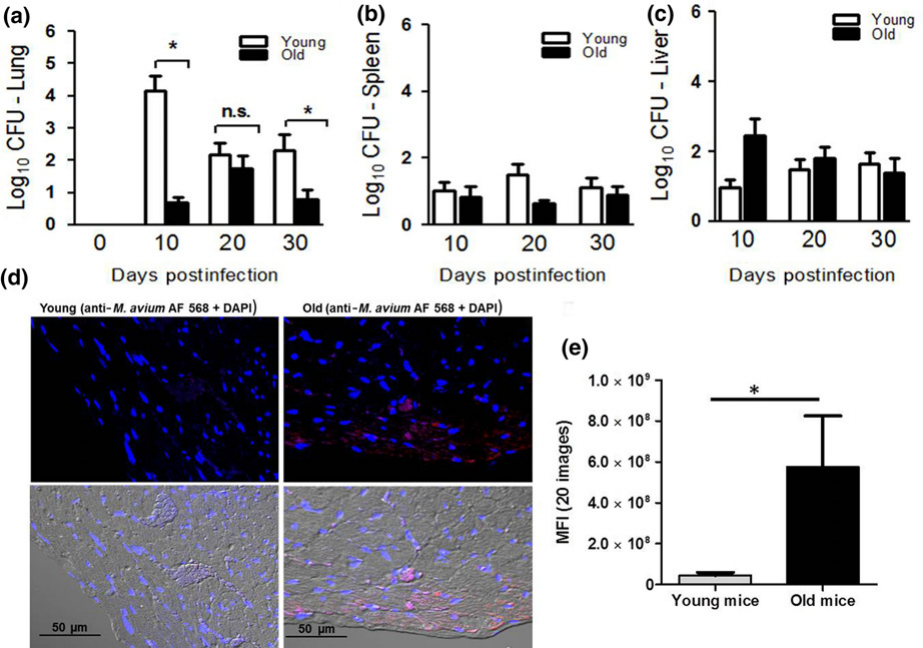
*Mycobacterium avium* bacterial burden in lungs of young and old mice. C57BLC57BL/66 mice intranasally infected with *M. avium* strain 104 (1.2 × 10^5^ CFU) were euthanized at 10, 20, and 30 days postinfection, and lungs were homogenized in sterile saline buffer. Serial lung (a), spleen (b), and liver (c) tissue dilutions were plated onto OADC supplemented 7H11 plates and incubated for 21 days at 37°C. CFU were counted and expressed as Log_10_ CFU; 4–5 mice per group per time point (mean ± *SEM*; *p < 0.05). The images shown in (d) are hearts of *M. avium* infected young and old mice, stained with anti‐*Mycobacterium tuberculosis* polyclonal antibody followed by anti‐rabbit Alexa flour 568 conjugated secondary antibody. The section was examined for *M. avium* by confocal microscopy. Images shown are representative of hearts from five young and old animals. (e) Randomly selected confocal images (5 images per heart, *n* = 5) from *M. avium* infected young and old mice hearts were analyzed by ImageJ, and the mean fluorescent intensities were plotted in the graph (mean ± *SEM*; *p < 0.05)

Intranasal infection with *M. avium *strain 104 (1.2 × 10^5^ CFU) resulted in detectable bacterial loads as early as 10 days postinfection in the lung, spleen, and liver homogenates of young and old mice. Compared to young mice, the lung CFU count in old mice was significantly lower at 10 days postinfection (p.i.) and remained significantly lower in the lungs of old mice through 30 days p.i (Figure 1a). The mycobacterial CFU load in the spleen and liver of old and young infected mice remained comparable at the measured time points (Figure 1b‐c). We also performed immunoflourescence microscopy on heart tissue sectioned from infected old and young mice 35 days p.i. Notably, *M. avium* infected old mice had disseminated mycobacteria in the superior region of the heart, particularly in the pericardial sac **(**Figure 1d), but the bacterial load in the heart was sublethal.

### 2.2 *M. avium* dissemination causes premature atrial contractions and cardiac hypertrophy in old mice

**Figure 2:**
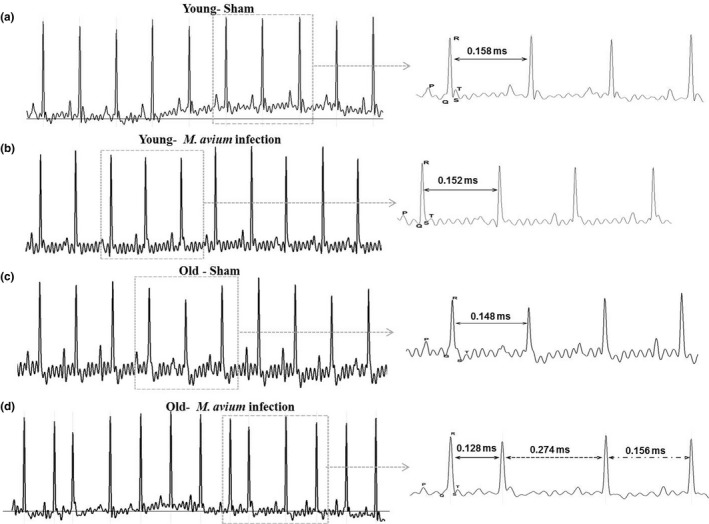
*Mycobacterium avium *infection causes cardiac arrhythmia in old mice. Surface electrocardiogram (ECG) recordings of control and *M. avium* (200 CFUs) infected young and old mice at baseline and at 30 days postinfection by PowerLab 4/30 (AD Instruments). ECG traces were analyzed using LabChart 8 Pro (AD Instruments). (a) Shown are representative ECG data from sham‐treated young mice (*N* = 5), (b) *M. avium* infected young mice (*N* = 5), (c) sham‐treated old mice, and (d) *M. avium *infected old mice at day 30 postinfection. Data shown are a representative of 5 mice (*N* = 5)

Since *M. avium* disseminated into the heart of old mice, we next examined the cardiac electrical activity of *M. avium* infected or aged matched control mice. The ECG data analysis indicated that *M. avium* infection did not cause alteration in cardiac electrophysiology in young mice (Figure 2a,b). Whereas in old mice, *M. avium* dissemination into the heart caused irregular RR intervals at every 4 peaks, indicative of sinus pause or premature atrial contractions (Figure 2d). These ECG abnormalities were not found in uninfected old mice or *M. avium *infected young mice (Figure 2a–c).

**Figure 3:**
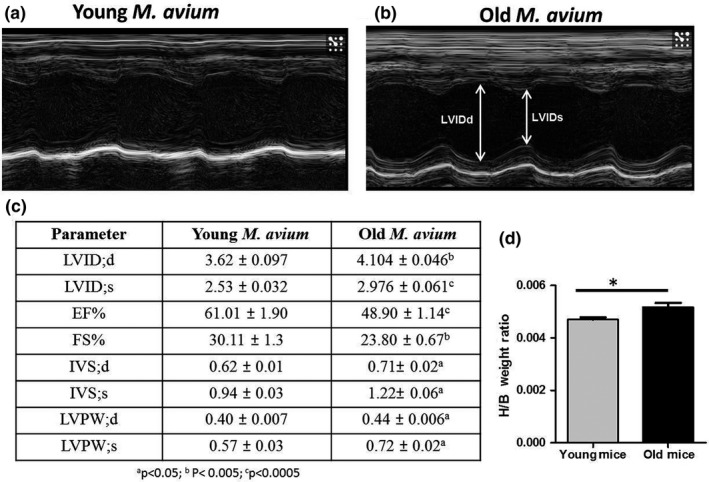
Hearts of *Mycobacterium avium *infected old mice undergo cardiac hypertrophy. To assess heart function in vivo, 2D‐echocardiography (Vevo 2100, Visualsonics) was performed in *M. avium* (200 CFUs) infected young and old mice at 30 days postinfection. Representative baseline M‐mode echocardiographs from *M. avium* infected young (a) and old (b) mice (*N* = 6). (c) Atleast two M‐mode echocardiogram measurements from each mouse were used to determine the LVID_d_, left ventricular end‐diastolic dimension; LVIDs, left ventricular end‐systolic dimension, ejection fraction (EF%), fractional shortening (FS%), IVST_d_, interventricular septal thickness in diastole; IVSTs, interventricular septal thickness in systole; LVPW_d_, posterior wall thickness in diastole; LVPWDs, posterior wall thickness in systole, ^a^ *p* < 0.05; ^b^ *p* < 0.005; ^c^ *p* < 0.0005. (d) Graph shows the heart weight over the body weight of young and old mice (baseline), 20 mice/group (mean ± *SEM*; *p < 0.05)

To determine whether there were multiple RR intervals among treatment groups, 10 min of ECG recordings was overlaid in 30‐s increments and analyzed using LabChart Pro‐8 software. *M. avium* infected old mice were found to have three distinct RR intervals (Supporting Information Figure S2C), while *M. avium* infected young mice and both young and old uninfected groups have similar RR interval (Supporting Information Figure S2A–C). We also determined the heart rate (HR) from recorded ECG data and compared the experimental groups. *M. avium* infected old mice showed significant reduction in HR as well as increased variability (236–400 bpm; Supporting Information Figure S2D). The HR in young mice was not affected by infection (Supporting Information Figure S2B–D). The heart function of sham treated and *M. avium* infected young and old mice was also assessed by echocardiography (Echo)(Figure 3a,b). Our results revealed that the heart function was normal in sham treated young and old mice (Figure S3D,B,C). *M. avium* infected old mice had thickening of the left ventricular walls (anterior wall in diastole and systole), an increase in left ventricular diameter in both diastole and systole, and deteriorating heart function as shown by decreased fractional shortening and ejection fraction **(**Figure 3b,c).

### 2.3 *M. avium* infection aggravates systemic inflammation in old mice

Since it is well established that aging is associated with chronic systemic inflammation, we questioned how infection with *M. avium* influenced the inflammatory state of old mice. We analyzed the serum of *M. avium* infected young and old mice 30 days p.i. for levels of pro‐inflammatory cytokines IL‐1β and TNF‐α. Consistent with previous studies regarding chronic systemic inflammation in mice (Starr, Saito, Evers, & Saito, 2015), the serum of old uninfected mice contained higher levels of IL‐1β and TNF‐α compared to uninfected young mice (Supporting Information Figure S1A,B). *M. avium* infected old mice had significantly higher levels of IL‐1β and TNF‐α compared to *M. avium* infected young mice (Supporting Information Figure S1A,B). The levels of IL‐1β and TNF‐α in *M. avium* infected old mice were roughly 2.5‐ to 3‐fold higher than in the age‐matched uninfected controls. Collectively, these results suggest that pulmonary NTM infection further aggravates systemic inflammation in old mice.

### 2.4 Enhanced immune cell infiltration in the heart of *M. avium* infected old mice

Mycobacteria disseminated into the heart of old mice, and these mice also showed abnormal ECGs and Echo. We questioned whether there was any leukocyte infiltration of into the cardiac muscle of infected mice. We stained heart sections (four chamber view) from *M. avium *infected old and young mice with αCD45, a common leukocyte marker, and visually analyzed for the presence of CD45^+^ cells. While *M. avium* infection increases the infiltration of immune cells in the heart of both young and old mice (Supporting Information Figure S4A‐D), the infiltration was significantly higher in old mice (Figure S4e–g). We particularly noted a substantial increase in the infiltration in the superior, ventricle, and septa region of the heart in *M. avium *infected old mice (Supporting Information Figure S4B,D).

### 2.5 *M. avium* dissemination induces cardiac fibrosis in old mice

**Figure 4:**
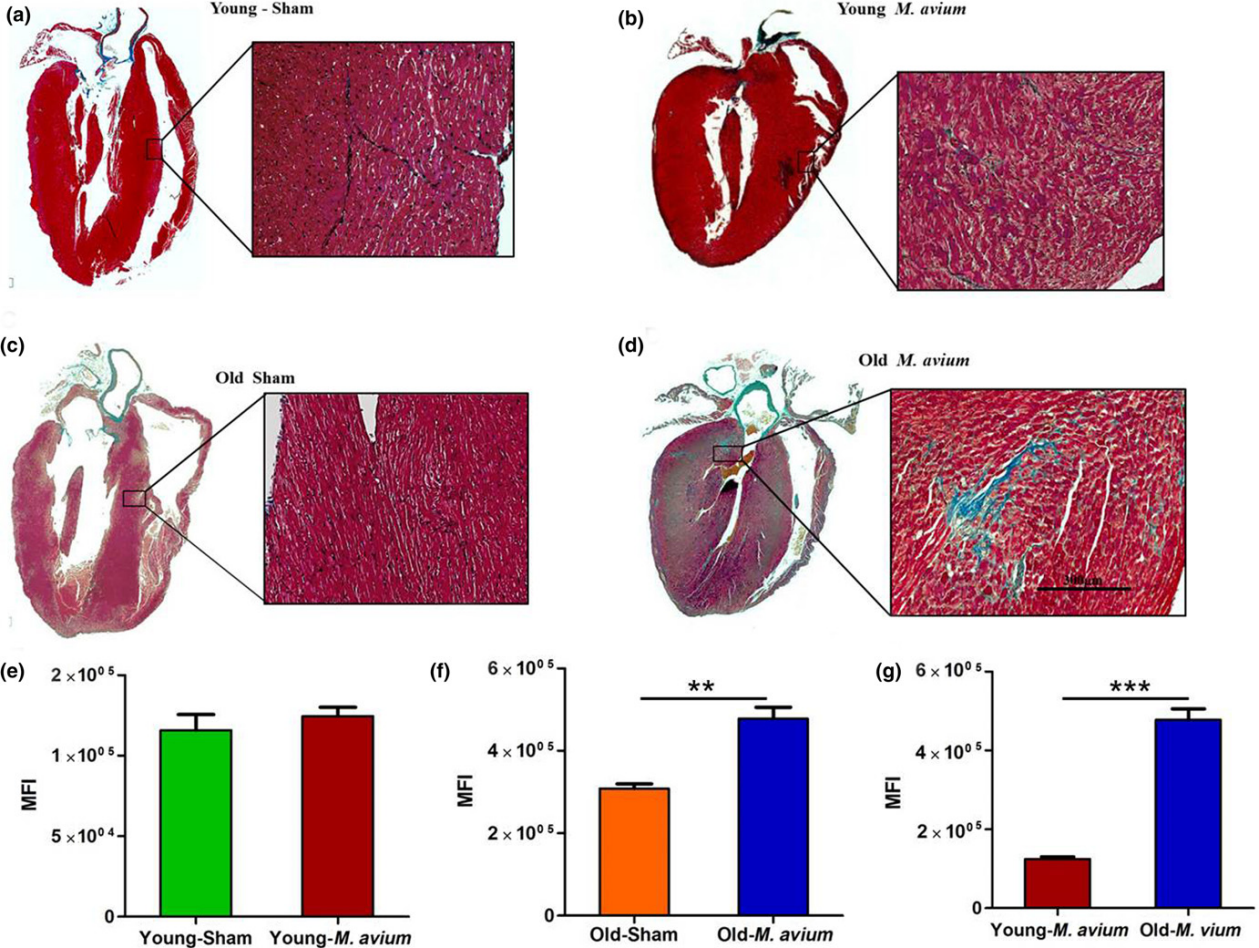
NTM infection induces cardiac fibrosis in old mice. Heart sections from sham and *Mycobacterium avium* infected young and old mice were stained with Masson's trichrome to identify fibrosis in cardiac tissue. Image shown here are representative of whole heart and interstitial fibrosis from sham‐treated young (a) and old (c) mice that were infected with *M. avium* (b and d). The image shown in right panel is 40× magnification and representative of five animals/group. The trichrome staining (blue color) in the heart sections was isolated using Photoshop CC in color range selection mode. The total intensity of the stained area in the heart sections was further quantified via ImageJ using color intensity to multiply area with staining. Graph shown in (e) young mice sham‐treated and *M. avium* infected, (f) old mice sham‐treated *M. avium* infected, and (g) comparison of *M. avium* infected young and old mice. Data shown in graphs are cumulative data from five animals (mean ± *SEM*; ** *p* < 0.005; ***p < 0.0005; *N* = 5)

Excessive immune cell infiltration and cell death in tissues are correlated with the development of tissue fibrosis (Biernacka & Frangogiannis, 2011; Kong, Christia, & Frangogiannis, 2014; Suthahar, Meijers, Sillje, & de Boer, 2017). We therefore assessed whether cardiac infiltration of immune cells during *M. avium* infection induces fibrosis in the cardiac tissue of *M. avium *infected old and young mice. In the uninfected groups, old mice had basally higher levels of fibrosis than young mice (Figure 4a,c,f). *M. avium* infection did not increase heart tissue fibrosis in young mice (Figure 4b,e), but significantly increased heart tissue fibrosis in old mice (Figure 4c,f,g). We additionally noted increased perivascular and interstitial fibrosis in *M. avium *infected old mice and significantly thickened left ventricles (Supporting Information Figure S5A–E).

### 2.6 Dissemination of *M. avium* triggers the inflammatory gene expression in the hearts of infected mice

**Figure 5:**
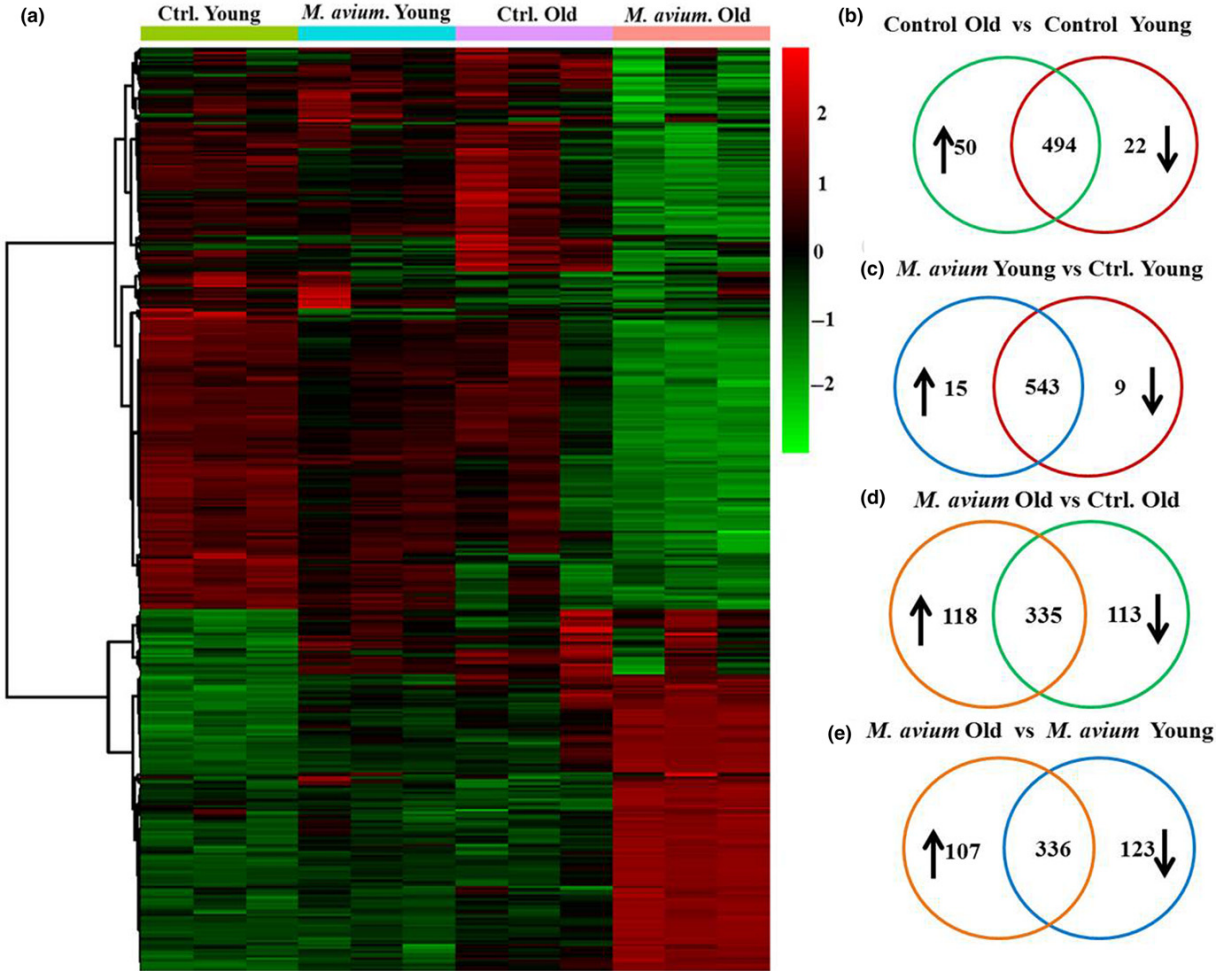
Changes of immune‐related gene expression in the hearts of *Mycobacterium avium* infected young and old mice. Hearts were collected from sham‐treated or *M. avium* infected (30 days postinfection) young and old mice to isolate mRNA. The mRNA samples were analyzed using NanoString assay on the mouse pan‐cancer immune panel, followed by data analysis in nSolver software. The heat map showing genes whose induction in heart tissue by *M. avium *infection was more than 1.5‐fold different in young and old mice (a), *p* < 0.05. The vein diagrams shown in (b‐e) is the number of genes that were upregulated, downregulated, or no change in expression in young and old mice that were infected or uninfected with *M. avium.* Two‐tailed Student's *t* test is used to select differentially expressed genes with values *p* < 0.05

To determine whether cardiac infiltration of immune cells causes cardiac inflammation, we examined the inflammatory gene expression in the hearts of *M. avium* infected animals and we compared the global mRNA expression in the heart of infected and uninfected mice by NanoString nCounter (Immunology panel) assay (Figure 5a). A cutoff of *p* < 0.05 was considered statistically significant, and the experimental groups were compared as followed, Control Old vs. Control Young (Figure 5b), *M. avium* Young vs. Control Young (Figure 5c), *M. avium* Old vs. Control Old (Figure 5d), and *M. avium* Old vs. *M. avium* Young (Figure 5e).

Independent of infection (Control Old vs. Control Young), there were 72 differentially expressed genes in old mice (Figure 5b and Supporting Information Table S1), when compared against uninfected young mice, we only noted 22 differentially expressed genes in the heart of *M. avium *infected young mice (*M. avium* Young vs. Control Young, Figure 5c). In contrast, when compared against uninfected old mice, *M. avium* infected old mice had 231 genes that were differentially expressed (118 up and 113 down; *M. avium* Old vs. Control Old, Figure 5d and Supporting Information Table S2). Lastly, when we compared the *M. avium* infected groups against each other (*M. avium* Old vs. *M. avium* Young), 230 genes were differentially expressed (107 up and 123 down; Figure 5e and Supporting Information Table S3).

**Figure 6:**
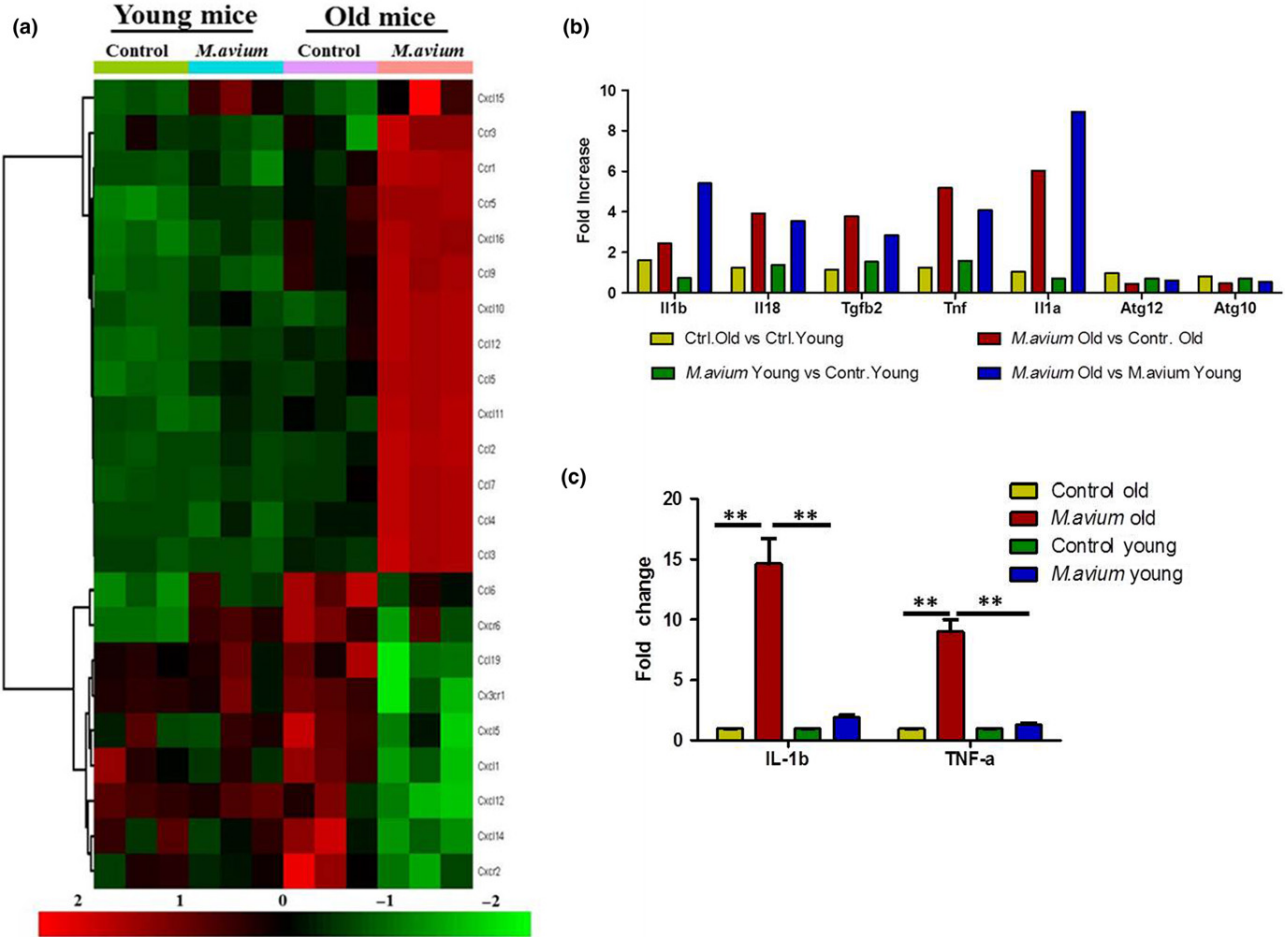
Comparative analysis of chemokine and chemokine receptors, inflammatory genes, and validation of NanoString data. (a) Heat map showing differential gene expression of chemokines and chemokine receptors in the heart tissue of uninfected and *M. avium *infected young and old mice. (b) Selected inflammatory gene expression of uninfected and *M. avium *infected young and old mice. (c) Validation of IL‐1β and TNF–α by qRT–PCR. The total RNA from heart tissue was reverse transcribed and used to determine the expression of IL‐1β and TNF–α by qRT–PCR using Taqman primers (*N* = 5). Data shown in graphs are cumulative data from five animals (mean ± *SEM*; **p < 0.005)

We also found an increased expression of many chemokines and chemokine receptors in the heart of *M. avium* infected old mice (Figure 6a). The inflammatory cytokines IL‐1β, TNF‐α, IL‐18, and IL‐1α were among the upregulated genes in *M. avium* infected old mice (Figure 6b). Consistent with previous findings regarding age‐associated defects in autophagy (Shirakabe, Ikeda, Sciarretta, Zablocki, & Sadoshima, 2016), we noted decreased expression of autophagy‐related genes (Atg10 and Atg12) in the hearts of *M. avium* infected old mice compared to young mice. In regard to cardiac fibrosis, we observed an increased expression of fibrosis‐inducing factor Tgfb2 in the heart of *M. avium* infected old mice, which correlates with an increased fibrosis in *M. avium* infected old mice (Figure 4d and Supporting Information Figure S4B). Together, these data strongly indicate that *M. avium* infection induces cardiac inflammation that leads to a defect in cardiac electrical activity and fibrosis.

### 2.7 Validation of mRNA expression patterns by qRT–PCR

To further validate the gene expression profiling results that we obtained from NanoString technology, we used qRT–PCR to assay expression of selected upregulated genes (TNF‐α and IL‐1β). The gene expression profiles of TNF‐α and IL‐1β obtained by NanoString immunoassay were in agreement with the gene profiles obtained by qRT–PCR (Figure 6c).

### 2.8 *M. avium* dissemination in old mice upregulates canonical pathways related to immune cell recruitment, inflammation, fibrosis, and cell death

Using Ingenuity Pathway Analysis (IPA), we examined the relationship between these differentially expressed and highly significant genes (230 genes) to determine the most significant canonical pathways and biological networks involved in *M. avium* infected old mice hearts (Table 1). The top enriched categories of canonical pathways (*p* < 0.05) were associated with granulocyte adhesion, macrophages, fibroblasts, and endothelial cell activation PRRs in recognition of bacteria and viruses, and cardiac hypertrophic signaling, apoptosis and death receptor signaling, and TGFβ signaling end of sentence.

**Table 1:** Top significantly enriched canonical pathways in *Mycobacterium avium *infected old animal hearts by IPA

Pathway	−log (*p*‐Value)	Ratio	Molecules
Granulocyte Adhesion and Diapedesis	32	0.188	Ccl2,CXCL12,ITGAM,IL1RL1,Ccl8,CSF3R,ITGB2,CCL3L3,THY1,Cxcl11,ITGA1,IL1RN,C5AR1,TNF,CXCL16,CCL4,ICAM2,Ccl7,IL1B,CCL5,TNFRSF1B,CXCL10,FPR2,CCL24,CCL21,CDH5,CCL19,IL18,Ccl9,IL1A,JAM3,PECAM1
Macrophages, Fibroblasts and Endothelial Cells in Rheumatoid Arthritis	28.1	0.119	IL1RL1,CXCL12,ATF2,FCGR1A,TLR1,SOCS3,TLR9,IL16,MAPKAPK2,FOS,TLR8,C5AR1,TNF,CREBBP,TLR6,TLR2,VEGFC,IRAK1,TLR7,IL1B,CCL5,TNFVEGFA,NFATC1,IL18,MYC,MAP2K4,CCL2,IL1A,MAPK1,SOCS1,FCGR3A/FCGR3B
Pattern Recognition Receptors in Recognition of Bacteria and Viruses	26	0.197	TLR1,TLR9,C3AR1,TLR8,IRF7,C5AR1,TNF,OAS3,TLR6,TLR2,PRKCE,TGFB3,TLR7,IL1B,CCL5,C1QB,OAS2,CASP1,TGFB2,DDX58,IL18,NLRP3,MAP2K4,IL1A,CLEC6A,MAPK1,IFNB1
Toll‐like Receptor Signaling	23.7	0.276	IL1RL1,IRAK1,SIGIRR,TLR7,IL1B,TLR1,CD14,TLR9,FOS,TLR8,IL18,IL1RN,TNF,MAP2K4,IL1A,TICAM2,MAPK1,TAB1,TLR6,TLR2,ECSIT
IL‐10 Signaling	19.8	0.261	IL1RL1,IL1B,SOCS3,CCR1,CD14,FCGR2A,FOS,CCR5,IL18,IL1RN,FCGR2B,TNF,MAP2K4,IL1A,MAPK1,IL4R,TAB1,IKBKE
p38 MAPK Signaling	19.1	0.175	IL1RL1,IRAK1,TGFB3,ATF2,MAP3K5,IL1B,TNFRSF1B,TGFB2,PLA2G6,FADD,MEF2C,MAPKAPK2,HSPB2,IL18,MYC,IL1RN,TNF,MAP2K4,IL1A,CREBBP,TAB1
IL‐6 Signaling	18.1	0.157	IL1RL1,IL1B,VEGFA,TNFRSF1B,CD14,TLR9,MAPKAPK2,IL6ST,HSPB2,IL18,PIK3CD,IL1RN,IL1R1,MAP2K4,TNF,MAP2K2,IL1A,SOCS1,MAPK8,HRAS,IKBK
NF‐κB Signaling	18.5	0.128	IRAK1,SIGIRR,PDGFRB,EGFR,TLR7,IL1B,KDR,TLR1,TNFRSF1B,TLR9,FCER1G,,IGF1R,TLR8,IL18,IL1RN,TNF,LCK,IL1A,CREBBP,TAB1,TLR6,TNFRSF11A,TLR2
Atherosclerosis Signaling	17.3	0.157	CXCL12,IL1B,COL3A1,ITGB2,MSR1,COL1A1,PLA2G6,CMA1,LYZ,IL18,CD36,IL1RN,TNF,CLU,CCL2,IL1A,SELPLG,CSF1,TNFRSF12A,CCR3
Fibrosis/Hepatic Stellate Cell Activation	24.8	0.155	CCR7,IL1RL1,COL1A1,SMAD4,A2M,CCR5,FN1,TNF,SMAD3,VEGFC,TGFB3,PDGFRB,COL4A1,EGFR,IL1B,CCL5,KDR,TNFRSF1B,CD14,VEGFA,COL3A1,TGFB2,CCL21,IGF1R,CCL2,BCL2,IL1A,CSF1,IL4R
Activation of IRF by Cytosolic Pattern Recognition Receptors	13	0.206	ATF2,STAT2,DDX58,IFIT2,IRF7,MAP2K4,TNF,ISG15,CREBBP,IFNB1,IKBKE
IL‐12 Signaling and Production in Macrophages	11.4	0.11	PRKCE,TGFB3,TGFB2,TLR9,MAF,PPARG,IL23R,FOS,LYZ,IL18,TNF,CLU,MAP2K4,MAPK1,TLR2,IKBKE
TGF‐β Signaling	8.38	0.118	FOS,TGFB3,IRF7,MAP2K4,BCL2,SMAD3,TGFB2,MAPK1,CREBBP,TAB1,SMAD4
HMGB1 Signaling	7.52	0.0863	FOS,TGFB3,IL18,IL1B,TNFRSF1B,TNF,MAP2K4,CCL2,IL1A,TGFB2,MAPK1,TLR9
Death Receptor Signaling	7.27	0.108	HSPB2,TNFRSF10A,MAP3K5,TNFRSF1B,TNF,MAP2K4,BCL2,FADD,BID,IKBKE
Chemokine Signaling	6.91	0.117	FOS,CCR5,CXCL12,CCL5,CCL2,CCL24,MAPK1,CCL4,CCR3
Apoptosis Signaling	6.08	0.0938	PRKCE,MAP3K5,TNFRSF1B,TNF,MAP2K4,BCL2,MAPK1,BID,IKBKE
Cardiac Hypertrophy Signaling	4.97	0.0498	IGF1R,TGFB3,ATF2,MAP3K5,MAP2K4,TGFB2,MAPK1,TLR9,CREBBP,TAB1,MEF2C,MAPKAPK2
IL‐17A Signaling in Fibroblasts	4.5	0.143	FOS,CCL2,MAPK1,LCN2,IKBKE

#### 2.8.1 Disease and function analysis and top upstream regulators

In addition to canonical pathways, the differentially expressed genes in *M. avium* infected old mice hearts were also categorized to related disease and functions, and upstream regulators (Table 2). We found that cellular movement, development, cellular growth and proliferation, cell‐to‐cell signaling and interaction and cellular function and maintenance were considerably activated with increased number of interacting molecules in *M. avium* infected old mice. Notably, young mice infected with *M. avium* showed only few molecules that were activated in the above listed functional pathways (Supporting Information Table S5). The IPA upstream functional analysis was used to predict the top upstream transcriptional regulators from differentially expressed genes in hearts of old mice infected with *M. avium*. An overlap *p*‐value was added based on significant overlap between genes in the data set and known targets regulated by transcriptional regulators. IPA predicted top transcriptional regulators that were activated in our data set are TGFB1 (*p* = 3.77E‐61), TNF (*p* = 5.23E‐77), IFNɣ (*p* = 4.85E‐92), LPS (*p* = 2.19E‐103), and IL‐1β (*p* = 1.58E‐59). Whereas in young mice infected with *M. avium*, IPA predicted only few regulators that were activated such as IFNɣ (*p* = 1.13E‐09), LPS (*p* = 7.63E‐11), and STAT1 (*p* = 1.12E‐09).

**Table 2:** Top cardiac toxicity molecules by IPA

Ingenuity Toxicity Lists	−log (*p*‐value)	Ratio	Molecules
Cardiac Necrosis/Cell Death	13	0.0782	IRAK1,TXNIP,MAP3K5,IL1B,SOCS3,VEGFA,CASP1,FADD,LCN2,MAPKAPK2,IL6ST,DPP4,IL1RN,THBD,TNF,MAP2K4,BCL2,RRAD,MAPK1,ANGPT1,SPP1,CYBB,TLR2
TGF‐β Signaling	8.23	0.115	FOS,TGFB3,IRF7,MAP2K4,BCL2,SMAD3,TGFB2,MAPK1,CREBBP,TAB1,SMAD4
Cardiac Fibrosis	7.99	0.0698	MAP3K5,IL1B,VEGFA,EGR1,IL16,IGF1R,FN1,TNF,NLRP3,SMAD3,ETS1,SPP1,CYBB,TLR2,IKBKE
Cardiac Hypertrophy	7.37	0.0511	Ccl2,IL1RL1,CXCL12,HCK,EGFR,MAP3K5,IL1B,SMAD4,MEF2C,IL6ST,IL18,FN1,TNF,MAP2K4,RRAD,MAPK1,TAB1,NT5E
Increases Cardiac Dysfunction	3.51	0.0893	Ccl2,CD36,MAP3K5,TNF,CYBB
Increases Cardiac Dysfunction	2.87	0.0357	CCR2,CYBB
Increases Damage of Mitochondria	2.24	0.182	IL1B,TNF
Increases Cardiac Proliferation	1.8	0.06	EGFR,TNF,ITGB2
Increases Heart Failure	1.55	0.08	TNF,CASP1
Increases Cardiac Dilation	1.17	0.05	MAP3K5,TNF
TGF‐β Signaling	1.05	0.0104	FOS
Cardiac Fibrosis	0.723	0.00465	CYBB
Cardiac Necrosis/Cell Death	0.603	0.0034	CYBB

#### 2.8.2 Analysis for cardiotoxicity

To further identify the key pathways that are involved in the cardiac dysfunction, we examined the cardiotoxicity functions of differentially expressed gene sets. IPA analysis data reveal that gene network pathways linking to cardiac toxicity such as cardiac infarction, cardiac necrosis/cell death, cardiac fibrosis, inflammation, dysfunction, and enlargement are activated in *M. avium* infected old mice. In contrast, young mice infected *M. avium* showed very poor linkage with pathways connected cardiotoxicity (Table 2).

## 3 DISCUSSION

Bacterial infections and bacteremia significantly escalate the risk of cardiac failure in the elderly (Blecker et al., 2013; Court et al., 2002; Parrillo et al., 1990; Rudiger & Singer, 2007), and development of effective therapeutic interventions relies on comprehensive understanding of host–pathogen interactions. With the exception of epidemiological and case studies (Cordioli et al., 2015; Prevots & Marras, 2015), basic scientific information regarding cardiac dysfunction during mycobacterial infections is sparse. We are the first to show that the opportunistic pathogen *M. avium*, which is more closely associated with pulmonary and gastrointestinal colonization (Appelberg, 2006; Bermudez et al., 2000; Reddy, 1998), incurs significant cardiac damage and dysfunction in infected old mice.

The strain of *M. avium* (strain 104) used in this study was originally isolated from an AIDS patient, and its virulence and immunopathology have been previously characterized (Saunders, Dane, Briscoe, & Britton, 2002; Torrelles et al., 2002). As little as 100 CFU (via aerosol delivery) or a many as 5 × 10^7^ CFU (intraperitoneal injections) have been previously used to model chronic infection and immune responses (Saunders et al., 2002; Torrelles et al., 2002). The mild dose (1.2 × 10^5^ CFU) used in our experiments resulted in detectable CFU in surveyed organs (lung, liver, and spleen), with no apparent differences in pathogen‐specific adaptive immune responses (CD4^+^ T cell count, levels of INFɣ in lung homogenate; data not shown) in *M. avium* infected young and old mice. We also found that bacterial burden in the lung is decreased in old mice, while no difference in spleen and liver which may be due to the difference in circulating antimycobacterial antibodies in young and old mice. Since it has been shown earlier that containment of *M. avium* infection was due to increased levels of circulatory antimycobacterial antibodies (Huag et al., 2013). Nevertheless, *M. avium* dissemination aggravated the systemic levels of IL‐1β and TNF‐α in old mice. Secretion of IL‐1β is consequential of the activation of inflammatory cascades (inflammasomes), host triggered innate immune responses against invading bacteria (PAMPs), or damaged host proteins (DAMPs; Feldman, Rotter‐Maskowitz, & Okun, 2015; Guo, Callaway, & Ting, 2015; Rea et al., 2018). On the other hand, TNF‐α can be produced by a myriad of cells including antigen presenting cells, CD4^+^ T cells, fibroblasts, and epithelial cells, and mediates apoptotic and pro‐inflammatory cell signaling via transcription factor NF‐κB (Rea et al., 2018).

Cardiac hypertrophy is complex and influenced by genetic, physiological, and environmental factors involving particular transcriptional factors and contractile proteins (Hunter & Chien, 1999). Although cardiac hypertrophy is the heart's primary response to stress and an adaptive mechanism, prolonged stimulation of hypertrophy has adverse consequences that are linked to heart failure and sudden death (Braunwald & Bristow, 2000). Pleiotropic pro‐inflammatory cytokines like IL‐1β and TNF‐α aggravate endothelial cells, cardiomyocytes, fibroblast, and leukocytes into activated states. The activated endothelium enhances recruitment of circulating leukocytes and impacts cardiac fibroblast activity favoring the production of collagen (Biernacka & Frangogiannis, 2011; Kong et al., 2014; Suthahar et al., 2017). Excess collagen impacts the ability of cardio myocytes to propagate the cardiac action potential generated by the sinoatrial node, ultimately leading to abnormal diastolic function, and cardiac failure (Nguyen, Kiriazis, Gao, & Du, 2017; Nguyen, Qu, & Weiss, 2014). Cardiac hypertrophy is typically associated with diastolic dysfunction. Due to experimental limitations, we were unable to assess the degree of diastolic dysfunction in *M. avium *infected young and old mice. However, the systolic abnormalities detected in the hearts of *M. avium *infected old mice may result in a dilated phenotype and warrants further detailed investigation.

Performing subsequent immunofluorescence analysis of tissue heart sections from *M. avium* infected old mice confirmed the presence of mycobacteria in the pericardial sac of aged hearts and this was not detected in the hearts of *M. avium *infected young mice. The abnormally high levels of circulating IL‐1β and TNF‐α in *M. avium *infected old mice probably resulted from the actions of the innate immune response to mycobacterial PAMPS, but was initiated and eventually perpetuated by host DAMPs, as old mice already had pre‐existing heightened levels of the pro‐inflammatory cytokines. This conclusion is corroborated by the differential expression of genes in the hearts of *M. avium* infected old mice, and their particular association to canonical pathways related to innate immune cell recruitment and adhesion, pathogen‐related receptor signaling, fibrosis, cardiac hypertrophy, and cardiac toxicity. Although we did not investigate the principal sources of the pre‐existing inflammation in old mice, we suspect that changes in microbiota of the specific pathogen free mice used in this study were a contributing factor to their pre‐existing inflammation. In fact, a recent study has highlighted the contributions of age‐related changes in intestinal permeability and shifting microbiota (dysbiosis), toward driving age‐associated inflammation (Thevaranjan et al., 2018). Thevaranjan and team showed that germ free old mice (as well as TNF‐KO) did not have increased levels of circulating pro‐inflammatory cytokines and PAMPS and were essentially resistant to age‐associated inflammation.

In summary, we show that increased inflammation and bacterial dissemination into the heart of *M. avium *infected old mice have a profound impact on cardiac function. Our findings highlight an understudied area in mycobacterial research, all the while supporting previous findings related to bacterial sepsis and cardiac damage. We were only able to assess the cardiac function of *M. avium* infected young and old female mice, of the C57BL/6 genetic background in this study. Future studies will be needed to further investigate the link between clinical consequences of *M. avium* and cardiac dysfunction and, we can also speculate that other mycobacteria including *M. tb* may also induce cardiac dysfunction.

## 4 EXPERIMENTAL PROCEDURES

### 4.1 *M. avium* infection of mice

*M. avium* strain 104 was provided by Dr. Andrea Cooper [3]. *M. avium* cultures were grown to mid‐log phase in proskauer beck medium and stored in 1 ml aliquots at −80°C. Aliquots were then diluted in phosphate buffered saline (Invitrogen, Carlsbad, CA, USA) to desired concentrations for intranasal infections. Specific pathogen free C57BL/6 mice were obtained from the National Institute on Aging and Charles River laboratories (Wilmington, MA, USA). All animal studies were conducted in accordance with the American Physiological Society Guiding Principles for Research Involving Animals and Human Beings, and approved by The Ohio State University Institutional Animal Care and Use Committee. The investigation conforms to the Guide for the Care and Use of Laboratory Animals published by the US National Institutes of Health (NIH Publication No. 85‐23, revised 1996). Young (3 months) and old (18 months) female C57BL/6 mice were sedated with isoflurane prior to intranasal infection with 1.2 × 10^5^ CFU of *M. avium *in 20 µl of PBS (10 µl per nare) or treated with PBS only (sham). Experiments were repeated at least 3 times (*n* = 3) with 4–5 mice per group, per time point.

### 4.2 Bacterial burden

Mice were euthanized via CO_2_ asphyxiation prior to organ isolation. The lung, liver, and spleen of young and old *M. avium* infected mice were excised and homogenized in sterile NaCl (Sigma, St. Louis, MO, USA). Homogenized organs were serially diluted and plated onto 150 mm × 20 mm sterile plates of OADC supplemented 7H11 agar and incubated for 21 days at 37°C. *M. avium *colony‐forming units (CFU) were counted and expressed as Log_10_ CFU per organ, 4–5 mice per group per time point, repeated 3 times (*n* = 3).

### 4.3 Echocardiography

To assess cardiac function in vivo, 2D‐echocardiography (Vevo 2100, Visualsonics) was performed in *M. avium* infected young and old mice at 30‐day postinfection. Mice were anesthetized in an induction chamber at 2% isoflurane in oxygen at a flow rate of 1.0 L/min. Mice were then placed in supine position on a heated stage and hair was removed from the chest using depilatory lotion. Anesthesia was maintained at 1.5% isoflurane for the duration of the experiment. Heart rate was monitored throughout to ensure proper anesthetic dosage. Using a MS‐400 transducer, proper anatomical orientation was determined via imaging of the long axis of the heart. Once proper orientation was achieved, the transducer was turned 90 degrees to visualize the short axis of the left ventricle. M‐mode images were recorded at the level of the papillary muscles. Images were analyzed to assess ejection fraction, fractional shortening, chamber diameters, and left ventricular wall thicknesses.

### 4.4 Electrocardiography recordings

At day 35 post‐*M. avium *infection, subsurface electrocardiogram (ECG) was recorded. Young and old *M. avium* infected mice and aged matched control (sham) mice were anesthetized using 2% isoflurane in oxygen (flow rate 1.0 L/min), which was subsequently lowered to 1% (1.0 L/min) for the duration of the ECG recordings. Mice were placed in the prone position and kept on a heated pad to maintain body temperature. The subcutaneous electrodes for ECG were placed in the lead II configuration and ECGs were recorded for 10 min on a PowerLab 4/30 (AD Instruments, Houston, TX; Makara et al., 2016). ECG traces were analyzed using LabChart 8 Pro (AD Instruments).

### 4.5 Pathological methods and immunohistochemistry

At day 35 postinfection, the hearts of young and old *M. avium* infected mice and age‐matched uninfected controls were isolated and washed in cold PBS and then fixed in 10% formalin overnight at 4°C. The hearts were then switched to 20% sucrose solution. The fixed hearts were embedded in paraffin and sectioned for further analysis. To examine leukocyte infiltration, the four chamber view heart sections from *M*. *avium* infected and uninfected controls were stained with αCD45 antibody (Abcam, clone EP322Y). The heart tissue sections were deparaffinized and antigen epitopes were retrieved via heat‐induced antigen retrieval prior to incubation with αCD45 and biotinylated α‐rat antibody for 60 and 30 min, respectively. The numbers of αCD45+ cells in the heart sections of *M. avium* infected and uninfected controls (5 heart sections per group) were quantified using Aperio Image Scope software and converted to number of cells per µm^2^. To examine bacterial dissemination in the heart, heart sections were incubated with anti‐*Mycobacterium tuberculosis* polyclonal antibody (ab905; Abcam, Cambridge, MA, USA) followed by anti‐rabbit Alexa fluor 568 secondary antibody. The nuclei were stained with DAPI. To examine tissue fibrosis, heart sections from *M. avium* infected young and old mice and uninfected controls were stained with Masson's trichrome stain (Schipke et al., 2017). Trichrome stained heart sections were imaged, and positive staining (indicated by blue color) was quantified using Photoshop CC color range selection mode and ImageJ software. Trichrome color intensity was multiplied by measured areas to determine the mean fluorescent intensity (MFI) of heart sections (5 heart sections per group).

### 4.6 Cytokine assay

Blood samples from *M. avium* infected young and old mice and uninfected controls were collected immediately after euthanization. After a 1 hr incubation at 4°C, the blood samples were centrifuged for 10 min at 5,000 *g*. The clear supernatant was retrieved and stored at −80°C. The serum levels of cytokines IL‐1β and TNF‐α were determined by ELISA (5 samples per group) using commercially available kits (R&D Systems, Minneapolis, MN, USA). Values are represented as mean levels of cytokine ± *SEM*.

### 4.7 RNA extraction from isolated hearts

At day 35 postinfection, the hearts of *M. avium* infected young and old mice, and age‐matched uninfected controls were immediately isolated after euthanasia, rinsed in PBS to remove residual blood, and stored in RNAlater (Invitrogen) until RNA extraction. Briefly, isolated hearts (3 hearts per group) were homogenized in Trizol, after which chloroform was added and samples were centrifuged for 15 min at 12,000 *g*. The resulting aqueous phase was retrieved and RNA was precipitated by addition of isopropanol. Retrieved RNA was treated with DNAse1 and further purified using commercially available RNeasy kits (Qiagen) (Makara et al., 2016).

#### 4.7.1 NanoString nCounter assay

To investigate the gene expression in the hearts of *M. avium* infected young and old mice and age‐matched uninfected controls, we performed the NanoString nCounter assay (NanoString Technologies, Seattle, WA) using the mouse pan‐cancer immunology panel. All procedures were performed according to the manufacturer's protocol (NanoString Technologies). Briefly, 200 ng of high‐quality heart tissue RNA (3 mice per group) was hybridized to NanoString probes by incubation at 65°C for 18 hr. The hybridized RNA‐probe complexes were immobilized on a streptavidin‐coated cartridge using the nCounter Prep Station. The nCounter Digital Analyzer was used to count individual fluorescent barcodes and quantify target mRNA molecules.

#### 4.7.2 NanoString data analysis

Data normalization and analysis were performed by The Ohio State University Comprehensive Cancer Center—Share Genomic Facility and Bioinformatics Core Facility according to the manufacturer's guidelines. Specific background correction factors were applied to certain mRNAs according to the manufacturer's directions to account for nonhybridization‐dependent interactions of some bridge oligomers or capture and reporter probes. Technical normalization of the code counts was performed using spiked mRNA^+^ controls according to the manufacturer's instructions, and background was determined by the included negative controls. Each sample was then normalized to the geometric mean of the top 50 most highly expressed genes. mRNAs with normalized counts <100 (average background count) in all groups were removed, and the fold changes were calculated using the average of each group. For each experiment, the fold changes were calculated comparing the experimental group to their appropriate controls. Based on the normalized gene expression levels of NanoString‐based chips, two‐tailed Student's *t* test assuming equal variance was applied to each gene to compare the difference between the *M. avium* infected group and the sham‐treated group. Fold change cutoffs of >1.5 were used to evaluate gene expression changes with number. Calculations were performed using the R statistical computing environment. Bioinformatic analysis was performed to analyze differentially expressed genes in *M. avium* infected young and old mice. Briefly, outcomes form nanoString data analysis were first uploaded into Qiagens’ IPA system for core analysis and then with the global molecular network in the Ingenuity Pathway Knowledge Base (IPKB). IPA was performed to identify canonical pathways, disease and functions, and gene network that is related to cardiovascular diseases.

#### 4.7.3 NanoString data validation

The 100 ng of total heart RNA was reverse transcribed to cDNA by reverse transcriptase enzyme (SuperScript III; Invitrogen), and qRT–PCR was performed using mouse IL‐1β or TNF‐α TaqMan gene expression kit (Applied Biosystems). IL‐1β and TNF amplification was normalized to β actin as a housekeeping gene for relative gene expression. Triplicate samples were analyzed in duplicate wells in each experiment. (*n* = 3).

### 4.8 Statistical analysis

Statistical analysis was carried our using student *t* test or 2‐way ANOVA with the Tukey post hoc test, when appropriate. The results are shown as the Mean ± *SEM* values of *p* < 0.05 were considered to be significant.

## CONFLICT OF INTEREST

None declared.

## AUTHOR CONTRIBUTIONS

M.V.S.R. conceived the project and designed the experiments. M.V.S.R, C.H, A.G, S.M, and Q.W performed the experiments. P.F performed the NanoString assay, M.K analyzed the Echo data, L.P.G quantified the fibrosis, and L.Y analyzed the NanoString data and performed IPA analysis. M.V.S.R., C.H, M.K, L.P.G, and J.T analyzed and discussed the results, and reviewed the manuscript. M.V.S.R. and C.H wrote the manuscript with input from M.K, L.P.G, and J.T, and C.H and M.V.S.R prepared the figures.
