# Supplementary material for: Nontuberculous mycobacterium M. avium infection predisposes aged mice to cardiac abnormalities and inflammation
Source: Aging Cell. 2019 Mar 4;18(3):e12926. doi: 10.1111/acel.12926 (PMC6516181; doi:10.1111/acel.12926)
Supplement: Supplementary file 1 [file ACEL-18-e12926-s001.pdf]

Supplemental Figure 1: ***M. avium*** **infection enhances the serum cytokine levels in old mice.** The serum of uninfected and *M. avium* (1.2 x 10^5 CFU) infected young and old mice were analyzed for levels of pro-inflammatory cytokines IL-1β (**A**) and TNFα (**B**) via ELISA (mean ± SEM ; ***p<0.0005; N=5)

Supplemental Figure 2: ***M avium*** **infection causes premature atrial contractions in old mice.** The RR intervals (A & C) and heart rate (B &D) was determined from 10 minutes of recorded ECG from sham and *M. avium* infected young and old mice (N=4) by using Lab Chart 8 pro software (AD Instrument). In aged mice, *M. avium* infection shows variable RR intervals and heart rate, and decreased depolarization. Graph shown is a representative of 5 mice (N=2).

Supplemental Figure 3: Echo cartographic analysis of young and old mice. To assess heart function *in vivo*, 2D-echocardiography (Vevo 2100, Visualsonics) was performed in uninfected young and old mice. Representative baseline M-mode echocardiographs from young (A) and old (B) (N=5). (C) Atleast two M-mode echocardiogram measurements from each mouse were used to determine the $LVID_d$, left ventricular end-diastolic dimension; LVIDs, left ventricular end-systolic dimension, ejection fraction (EF%), fractional shortening (FS%), $IVST_d$, interventricular septal thickness in diastole; IVSTs, interventricular septal thickness in systole; $LVPW_d$, posterior wall thickness in diastole; LVPWDs, posterior wall thickness in systole.

Supplemental Figure 4: ***M. avium*** **infection enhances the infiltration of immune cells in to heart tissue of old mice.** Four chamber view sections from uninfected (A&C) and *M. avium* -infected (B &D) young and old mice were stained with CD45 antibody which indicates the infiltration of leukocytes in the heart. Photomicrographs of sham treated (A&C) and *M. avium* infected (B&D) septa region were shown with 10x and 40x magnifications. The graph shows the number of CD45 cells /100μm^2 of heart tissue from sham treated or *M. avium* infected young (E)

or old mice (F). Graph shown in (G) is comparison of CD45 positive cells in young and old mice infected with *M. avium*. The CD45 cells were counted from 10 randomly selected images from heart sections. Data shown in graphs are cumulative data from 5 animals, (mean ± SEM; ** $p<0.005$).

Supplemental Figure 5: *M.avium* infection enhances the cardiac fibrosis in old mice. Cross sections of hearts from *M. avium* -infected young and old mice were stained with Masson's Trichrome to identify fibrosis in cardiac tissue. Image shown here are representative of cross section whole heart young (A) and old (B) mice that were infected with *M.avium*. The image shown in right panel is 20x magnification and representative of 5 animals/group. The Trichrome staining (blue color) in the heart sections was isolated using Photoshop CC in color range selection mode. The total intensity of the stained area in the heart sections was further quantified via ImageJ using color intensity to multiply area with staining. Graph shown in (C) young and old mice infected with *M. avium*. Data shown in graphs are cumulative data from 5 animals, (* $p<0.005$) (N=5). (D) The left ventricular wall thickness in the heart sections were measured using the Aperio Image scope software. (* $p<0.005$) (N=5). (E) The cardiac cross sectional area of *M. avium* infected young and old mice was measured using Aperio Image scope software under 0.5x magnification (mean ± SEM; ** $p<0.005$; N=5)

Supplemental Figure 1

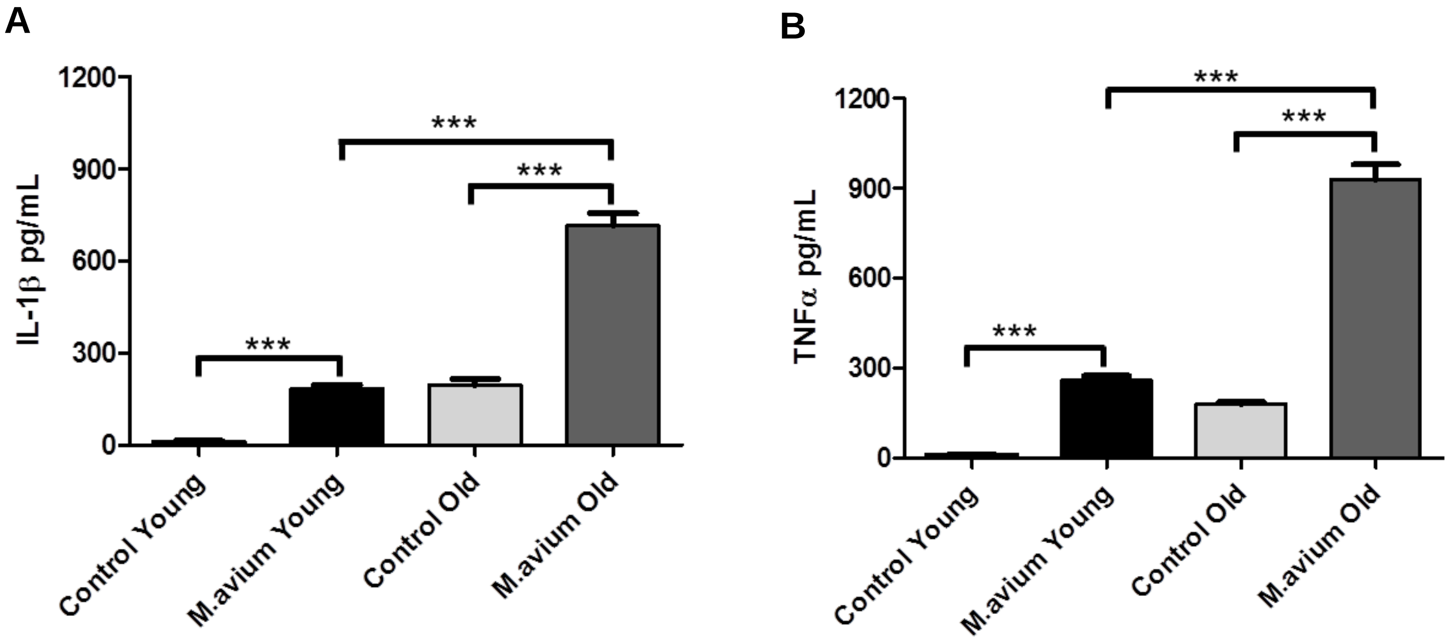

Supplemental Figure 2

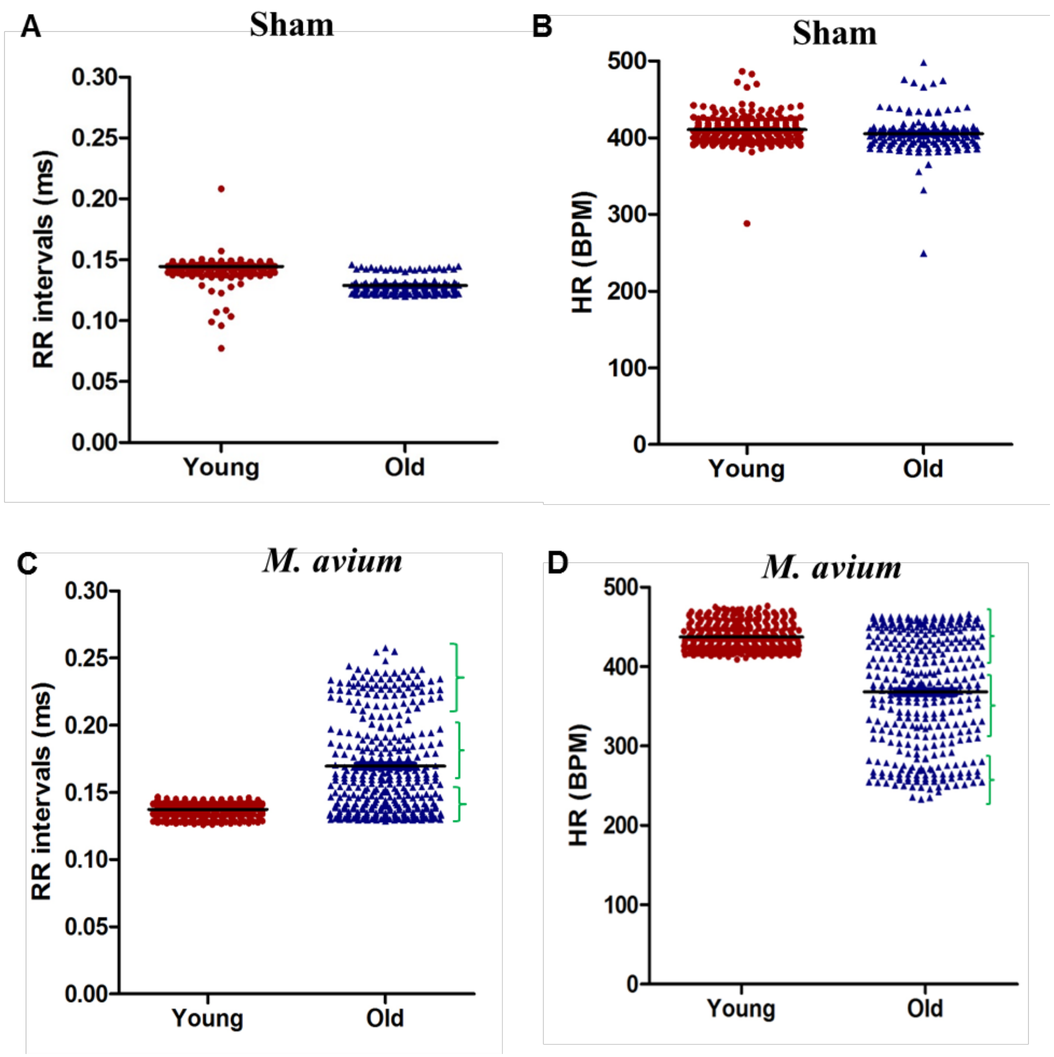

Supplemental Figure 3

A

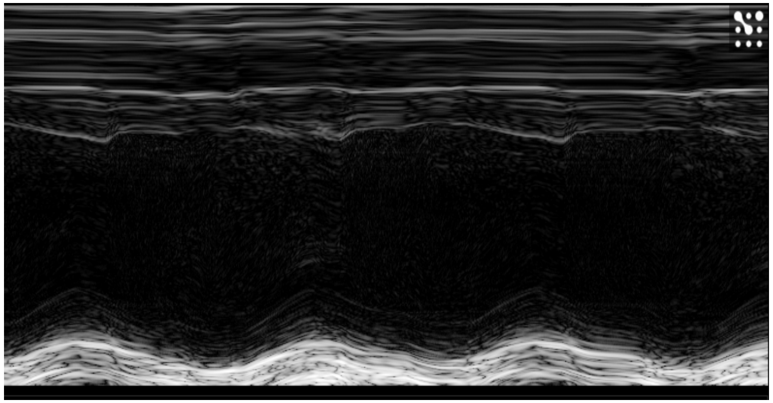

B

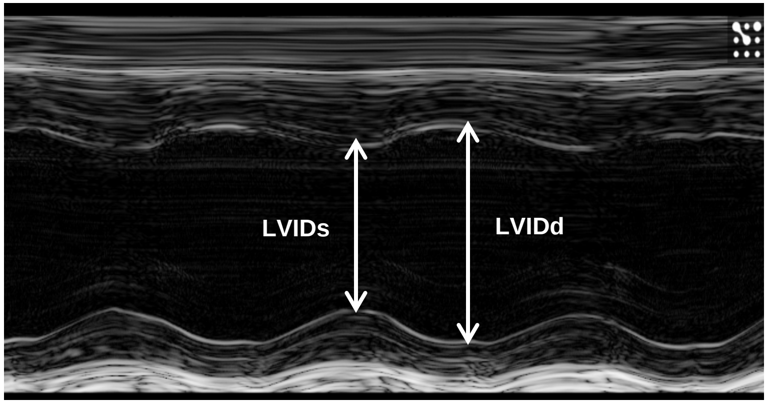

C

Parameter	Young	Old
LVID;d	3.98 ± 0.079	3.941 ± 0.130[ns]
LVID;s	2.71 ± 0.093	2.73 ± 0.064[ns]
EF	60.65 ± 1.78	58.35 ± 2.17[ns]
FS%	32.01 ± 1.23	30.45 ± 1.55[ns]
IVS;d	0.77 ± 0.083	0.85 ± 0.049[ns]
IVS;s	1.24 ± 0.080	1.25 ± 0.053[ns]
LVPW;d	0.537 ± 0.043	0.61 ± 0.0193[ns]
LVPW;s	0.88 ± 0.073	0.96 ± 0.023[ns]

Supplemental Figure 4

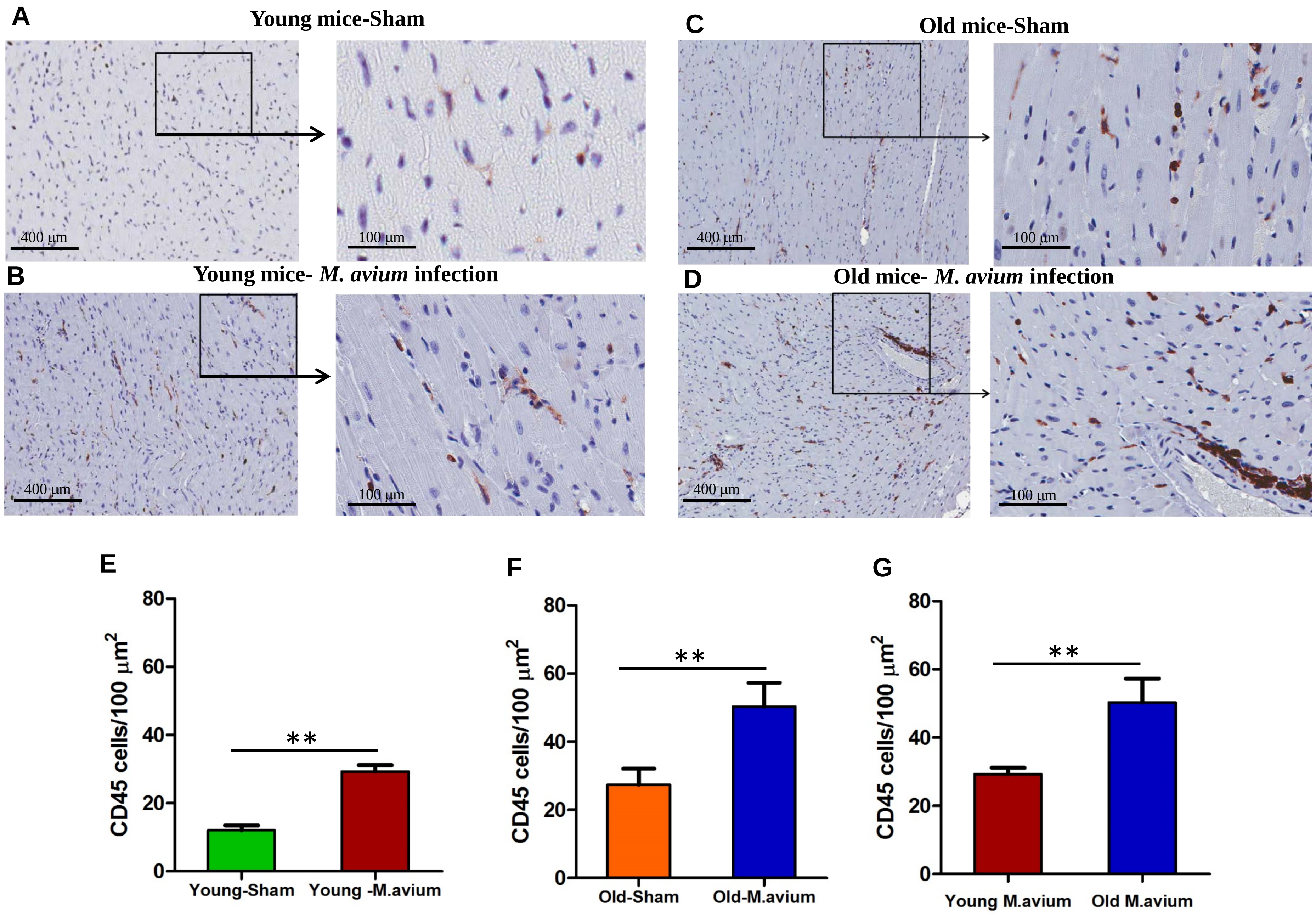

Supplemental Figure 5

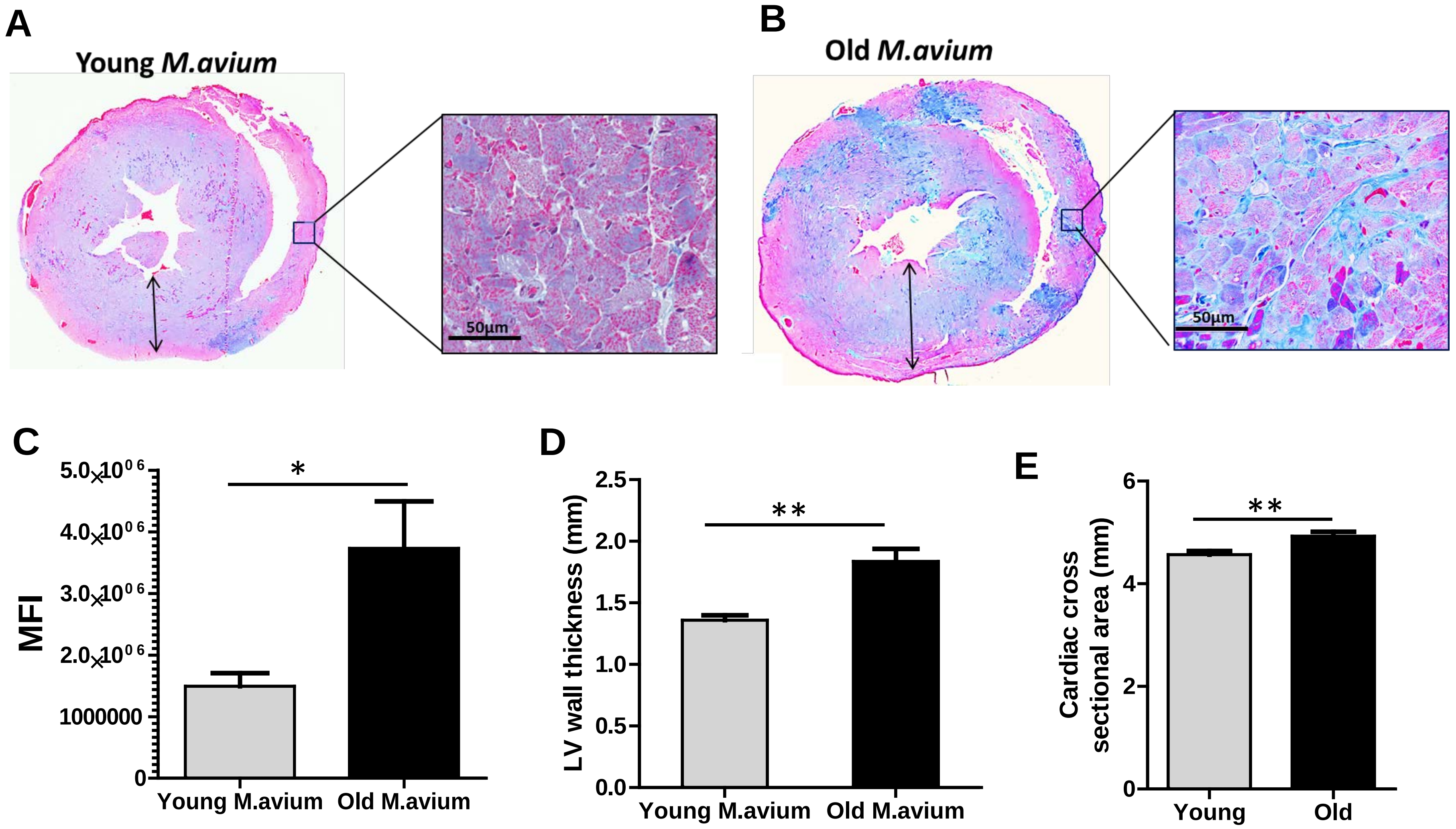

Number	Gene	Fold change M. avium Young vs Control Young	p. Value
1	Gbp2b	8.156216099	0.002554875
2	Ccl8	3.668016173	0.007050465
3	Ccl5	3.426415242	0.000181647
4	Zbp1	3.08656035	0.007761389
5	Nlrc5	2.897884309	0.003292765
6	H2-Aa	2.866916726	0.005708321
7	C4b	2.66456495	0.001997975
8	Klrk1	2.616611977	0.000122996
9	Ccr2	2.326078846	0.000788008
10	H2-K1	2.306811309	0.002043211
11	Klra2	2.244198051	0.007732149
12	Gzma	2.241710536	0.004469356
13	Slamf7	2.239536221	0.000236464
14	Cxcr6	2.11608855	0.005699367
15	Cybb	2.014190307	0.000407557
16	Ccl25	0.491444362	0.005350655
17	Tfrc	0.3900957	3.34E-05
18	Zap70	0.389879445	0.00093628
19	Cd19	0.311585499	0.004053236
20	Fos	0.277854209	0.000260981
21	Cd22	0.169317148	0.000502142
22	Dmbt1	0.047907655	3.41E-07

Supplementary Table1. **Immune profiling gene panel in heart tissue from young mice exposed to *M.avium*.** Total RNA purified from sham or M.avium infected young mice hearts were used for NanoString analysis by using nCounter Mouse PanCancer Immune Profiling Panel. Total RNA (200ng) purified from the heart tissue and used for NanoString analysis. The relative expression of each transcript was normalized against the expression of the top Genes with counts > 100. Each value is the mean of biological triplicate and the ratios between means per target genes provides linear Fold change. Two-tailed Student's t test is used to select differentially expressed genes with values < 0.05.

Number	Gene	Fold change *M. avium* Old vs Control Old	p. Value
1	Il1rn	133.8524745	1.77E-09
2	Spp1	78.29780725	2.18E-12
3	Ccl5	57.5203281	4.36E-10
4	Cxcl10	41.27796664	3.94E-07
5	Ccl3	25.91292557	5.89E-11
6	Ccl2	25.3462428	4.58E-11
7	Ccl7	24.77477414	2.21E-09
8	Fcgr1	21.01576401	4.41E-09
9	Ccl4	17.57923031	1.98E-08
10	Cxcl11	17.54879421	6.16E-08
11	Cd180	17.12105858	3.51E-10
12	Marco	16.97335671	3.76E-05
13	Fpr2	15.99667324	8.11E-08
14	Slamf7	14.28669198	6.08E-10
15	Ccl12	14.12716428	1.85E-08
16	C3ar1	12.4226409	6.73E-12
17	Msr1	12.00280757	3.85E-10
18	Cmpk2	11.32469272	2.62E-09
19	Ifit2	10.92226379	1.42E-07
20	Cxcl15	10.73164997	2.08E-03
21	Cd69	10.15205581	1.90E-09
22	Fcgr4	10.12114075	2.59E-05
23	Lgals3	9.251581459	1.15E-08
24	Rsad2	9.121682785	4.97E-08
25	Tlr1	8.886405653	7.70E-09
26	Oasl1	8.838490519	9.23834E-07
27	Emr1	8.600973737	2.55E-10
28	Cd68	8.084171905	2.79E-11
29	Ccr3	7.863109799	1.55E-04
30	Ctss	7.719460374	2.88655E-08
31	Isg15	7.563216934	4.26E-06
32	Ly9	6.903366122	2.72E-08
33	Tnfrsf1b	6.582337498	8.89E-10
34	Fcer1g	6.51109415	6.60E-10
35	Il7r	6.510642851	2.20E-07
36	Irf7	6.179970708	4.31E-05
37	Il1a	6.036448499	6.75E-06
38	Ikbke	5.778920408	3.12E-08
39	Hck	5.627912896	1.88E-07
40	Itgam	5.590587887	5.81E-07

41	Ifi44	5.444879043	9.81E-06
42	Ifit3	5.387440385	8.12E-05
43	Mefv	5.332825052	4.93E-06
44	Ebi3	5.272546816	1.63E-08
45	Oas3	5.197435682	4.02E-05
46	Tnf	5.183763862	6.28E-07
47	Trem2	5.071455999	2.78E-06
48	Tlr8	4.836287466	4.56E-08
49	Ifnb1	4.564636572	0.00127552
50	Usp18	4.460483186	1.12534E-05
51	Cfb	4.417101548	7.91E-04
52	Cd84	4.410065245	1.36397E-07
53	Il23r	4.373838648	6.35E-05
54	Zbp1	4.313921727	1.34E-03
55	Slc11a1	4.294231594	6.30679E-07
56	Ccr1	4.223092947	3.65E-06
57	Cybb	4.150542738	3.57E-07
58	Cd200r1	4.13331696	1.18821E-07
59	Mx2	4.027263864	7.75E-05
60	Pou2f2	4.010549725	3.41E-04
61	Ifit1	3.940011935	0.000435588
62	Il18	3.928830697	8.04E-07
63	Casp1	3.863209724	5.52E-08
64	Csf3r	3.860532879	5.33E-06
65	Itgb2	3.796842093	6.25E-07
66	Il21r	3.770876757	5.78603E-05
67	Herc6	3.700191659	8.35E-06
68	Thbs1	3.65431247	1.74E-07
69	Tgfb2	3.458868102	3.42E-07
70	Tlr9	3.38792045	2.29E-06
71	Ccl9	3.38674649	1.78E-06
72	Cd274	3.379008548	2.13E-03
73	Raet1	3.238217747	2.93E-04
74	Siglec1	3.143779429	2.87E-05
75	Blnk	3.10609079	9.90E-06
76	Clec4a2	3.096202863	1.27E-06
77	Ncf4	3.067365319	1.18562E-05
78	Csf2rb	3.06014497	8.41E-05
79	Tnfrsf11a	3.059932864	9.62E-07
80	Cfp	3.051672197	5.85E-05
81	Tlr7	3.032273741	1.16647E-06

82	Bst2	2.989284066	1.09E-04
83	Il2rg	2.95099198	1.74122E-05
84	Abcg1	2.90613153	7.51E-07
85	Irf5	2.855216216	7.9006E-06
86	Lyz2	2.826075483	5.94E-06
87	Cd14	2.793550692	7.17E-06
88	Isg20	2.760634707	1.72E-04
89	Fcgr2b	2.717160902	5.48E-07
90	Ddx58	2.689429181	1.06092E-06
91	Cd48	2.687938258	8.70E-07
92	Il4ra	2.626424294	4.89E-06
93	Lcn2	2.623876833	3.12011E-06
94	Itgal	2.616430614	8.45E-05
95	Alcam	2.571125443	3.98E-06
96	Ccr5	2.520307909	1.29E-05
97	Pik3cd	2.461947505	7.25848E-06
98	Bst1	2.454110143	0.000511975
99	Il1b	2.449351795	0.000684114
100	Socs1	2.446976082	8.62E-04
101	Maf	2.390313755	6.46E-06
102	Stat2	2.380888403	0.001587534
103	Ptprc	2.241710536	0.000169124
104	Cxcl16	2.192535193	9.72E-06
105	Btk	2.154420705	8.96E-05
106	Nlrp3	2.112132	5.60E-03
107	Ccl6	0.499896039	0.002288981
108	Cxcr6	0.499341944	9.05E-03
109	Map2k4	0.498201061	0.000201815
110	Kdr	0.49768334	0.000745456
111	Ceacam1	0.492569772	0.000640361
112	Bcl10	0.49127407	0.000119972
113	Il7	0.490491486	0.002347583
114	Stat5b	0.488963947	0.000711871
115	Il11ra1	0.488692883	0.001618514
116	Cd83	0.488659011	0.001485925
117	Lyve1	0.487948232	2.92692E-05
118	Mapk8	0.487576332	0.000302186
119	Tie1	0.487339815	0.000662717
120	Sigirr	0.486698421	0.000597596
121	Tnfsf13b	0.486327474	0.006280554
122	Map3k7	0.486125258	0.000492961

123	Creb1	0.484913727	0.000318912
124	Cd81	0.482633512	1.6973E-05
125	Tnfsf12	0.481864693	8.8811E-05
126	Masp1	0.481630947	0.00154431
127	Cxcr2	0.479200062	0.001479693
128	Reps1	0.478635729	0.000202035
129	Fyn	0.475593202	0.000137154
130	Cd163	0.474703963	1.58E-04
131	Cfh	0.47440792	7.92669E-06
132	Gzma	0.473455258	0.007234022
134	C8g	0.470054518	0.003121692
135	Serping1	0.469891638	1.38225E-05
136	Mcam	0.46648416	8.37411E-06
137	Slc7a11	0.466257876	0.003662304
138	C1s1	0.46616093	2.45E-05
139	Mef2c	0.46577335	0.000124904
140	Cd9	0.464805808	3.62729E-05
141	Hras	0.463005103	0.00214174
142	Atg10	0.459615705	0.000231614
143	Prkce	0.459329071	0.000260895
144	Atg12	0.457898581	7.85E-05
145	Syt17	0.456947397	0.00843347
146	Cxcl1	0.45546118	4.66E-04
147	Cxcl12	0.450437861	0.000147828
148	Map2k2	0.449720327	0.000149174
149	Il6st	0.448475165	2.27107E-05
150	Cd36	0.448319762	0.000205412
151	Fcer1a	0.447915967	0.005013687
152	Egr1	0.446706762	4.08785E-05
153	Psma2	0.445408193	1.25E-04
154	Ikzf2	0.44260756	0.000661001
155	Yy1	0.442392858	1.25064E-05
156	Selplg	0.442055679	0.000415535
157	Itga1	0.44031259	5.16882E-05
158	Ptgs2	0.432028981	0.000669833
159	Cd3eap	0.431191308	1.25E-04
160	Thbd	0.430862666	3.63498E-05
161	Mme	0.430206134	0.001154899
162	Angpt2	0.429342234	4.25984E-06
163	Il1rl1	0.429282718	2.17E-03
164	Lck	0.429015001	3.02E-03

165	Dusp4	0.426790509	0.001651043
166	Ada	0.426199262	0.000646086
167	Smpd3	0.425343408	0.00067192
168	Psmb7	0.422024892	6.03E-05
169	Anp32b	0.420943935	4.53E-06
170	Cxcl5	0.420564797	2.20E-03
171	Mill2	0.420331651	4.3354E-06
172	Tnfsf18	0.419865746	1.32E-03
173	Irak1	0.417717647	5.65E-05
174	Clu	0.416763258	5.94902E-05
175	Il1r1	0.416243601	8.02E-06
176	Atf1	0.415897524	7.92298E-05
177	Hmgb1	0.412195311	2.53206E-05
178	Vegfa	0.411938251	1.96E-04
179	Twist1	0.408922684	7.63747E-05
180	F13a1	0.408497739	1.28E-06
181	Rrad	0.40762092	3.84E-04
182	Cd34	0.403796413	7.43251E-07
183	Adora2a	0.403097293	5.42E-05
184	Mapkapk2	0.401368694	1.26038E-05
185	Cx3cr1	0.400090978	0.000102758
186	Cd3g	0.399536718	0.001647518
187	Klra5	0.399398273	0.001949771
188	Psmd7	0.395623492	1.13165E-05
189	Il13ra1	0.395239763	2.20438E-05
190	Thy1	0.394118124	1.59E-05
191	Tmed1	0.393681275	6.43E-05
192	Atf2	0.391612847	1.48227E-05
193	Cxcl14	0.390745185	0.000687521
194	Ifi27	0.390203872	1.52E-03
195	Xbp1	0.3900957	1.2692E-05
196	Hspb2	0.388799967	5.11E-05
197	Ccl27a	0.387696603	1.07E-03
198	S100a8	0.386569575	0.003747308
199	Vegfc	0.385739823	3.05971E-05
200	Tgfb3	0.38315499	4.00044E-05
201	Tek	0.382147103	4.87536E-06
202	Cma1	0.382014684	0.001570741
203	Jam3	0.38151191	5.22789E-06
204	Ets1	0.380271046	1.69607E-06
205	Bcl2	0.380244688	0.000203203

206	Timd4	0.379560035	0.000857354
207	Zfp13	0.379113044	8.08E-06
208	Hsd11b1	0.376912096	5.70E-06
209	Ccl21a	0.376103073	2.00936E-06
210	Pou2af1	0.375087737	1.59E-03
211	Dll4	0.371491215	9.16296E-05
212	Pecam1	0.368925138	8.63E-07
213	C1qbp	0.365869232	1.36E-05
214	S100b	0.360632169	6.61E-04
215	Lilra5	0.359284848	1.21E-04
216	Icam2	0.359259945	1.10E-06
217	Nt5e	0.356902027	6.19E-06
218	Bmi1	0.354092944	3.5014E-05
219	Ccl19	0.349896466	1.29E-04
220	Cd200	0.343028417	5.34E-06
221	Fez1	0.34231585	1.10479E-05
222	Lag3	0.341320743	1.90015E-05
223	Ccl24	0.329854113	0.000265932
224	Gzmm	0.327939139	0.000248378
225	Ecsit	0.320967694	3.88777E-06
226	Hamp	0.30245659	0.000405649
227	Rps6	0.296766469	1.70145E-05
228	Cd3d	0.293452723	0.000120492
229	Cd207	0.273573425	5.66246E-05
230	Rorc	0.208121801	4.6044E-07
231	Tnfrsf12a	0.187582871	7.8487E-06
232	Kit	0.174911858	0.008110328

Supplementary Table 2. **Immune profiling gene panel in heart tissue from old mice exposed to *M.avium*.** Total RNA purified from sham or *M.avium* infected old mice hearts were used for NanoString analysis by using nCounter Mouse PanCancer Immune Profiling Panel. Total RNA (200ng) purified from the heart tissue and used for NanoString analysis. The relative expression of each transcript was normalized against the expression of the top Genes with counts > 100. Each value is the mean of biological triplicate and the ratios between means per target genes provides linear Fold change. Two-tailed Student's t test is used to select differentially expressed genes with values < 0.05.

Number	Gene	Fold change *M. avium* Old vs *M. avium* Young	p Value
1	Il1rn	204.2227814	6.58E-10
2	Spp1	170.5321568	2.87E-13
3	Ccl5	67.79937176	2.70E-10
4	Ccl3	42.83446012	1.02E-11
5	Ccl7	40.65892031	3.96E-10
6	Fcgr1	39.25759805	4.76E-10
7	Ccl4	34.84065883	1.59E-09
8	Cxcl10	34.19231028	6.99E-07
9	Ccl2	30.04806117	2.45E-11
10	Ccl12	27.76316232	1.27E-09
11	Cxcl11	25.88061508	1.41E-08
12	C3ar1	17.33841362	1.46E-12
13	Lgals3	16.35545511	7.73E-10
14	Slamf7	14.93473776	4.98E-10
15	Cmpk2	14.47007004	8.33E-10
16	Fpr2	14.17915841	1.35E-07
17	Msr1	14.00820309	1.86E-10
18	Ifit2	13.92205235	4.70E-08
19	Fcgr4	13.58930718	7.78E-06
20	Cd180	13.55543965	9.84E-10
21	Isg15	13.150083	3.10E-07
22	Trem2	12.56466238	1.94E-08
23	Oasl1	11.37504226	2.75E-07
24	Cd68	11.20755753	4.71E-12
25	Ctss	11.14712684	4.16E-09
26	Ccl8	10.34527435	7.40E-05
27	Emr1	10.26029282	9.82E-11
28	Rsad2	9.387233203	4.28E-08
29	Fcer1g	9.304960332	8.03E-11
30	Ccr3	9.270197015	7.70E-05
31	Il1a	8.944494808	8.23E-07
32	Il23r	8.335680608	1.61E-06
33	Irf7	8.087534716	1.11E-05
34	Ifit3	7.576859522	1.36E-05
35	Ccl9	7.237041276	7.46E-09
36	Tlr1	7.172121855	2.57E-08
37	Oas3	7.111234345	7.07E-06
38	Tlr8	7.067500107	3.66E-09
39	Ccr1	7.067010243	1.28E-07
40	Hck	6.788063214	5.84E-08

41	Mefv	6.490366738	1.52E-06
42	Il7r	6.44821568	2.33E-07
43	Cd69	6.42189911	2.53E-08
44	Cd84	6.088555535	1.42E-08
45	Cd200r1	6.07253963	7.40E-09
46	Tnfrsf1b	6.07253963	1.50E-09
47	Ifi44	5.919190958	5.98E-06
48	Ly9	5.908942663	7.16E-08
49	Ifit1	5.765716894	4.87E-05
50	Zbp1	5.465676224	0.000406421
51	Ebi3	5.441860598	1.31E-08
52	Il1b	5.412143155	1.63E-06
53	Casp1	5.262688486	5.01E-09
54	Usp18	5.189875751	4.16E-06
55	Cfb	4.968464304	0.0004263
56	Herc6	4.914347922	1.05E-06
57	Ikbke	4.640241848	1.44E-07
58	Bst2	4.545692203	4.51E-06
59	Slc11a1	4.457083534	4.77E-07
60	Itgam	4.423229198	2.83E-06
61	Clec4n	4.244810061	5.06E-07
62	Clec4a2	4.22836524	8.46E-08
63	Marco	4.142782272	0.008504904
64	Tnf	4.095955382	3.33E-06
65	Lyz2	4.061464403	2.32E-07
66	Ifnb1	4.051342344	0.002321705
67	Cybb	4.011661839	4.67E-07
68	Lcn2	3.98560849	5.81E-08
69	Mx2	3.968241973	8.56E-05
70	Tlr7	3.943563846	1.10E-07
71	Il21r	3.922844096	4.38E-05
72	Fcgr2b	3.891428905	1.67E-08
73	Ncf4	3.756268002	2.10E-06
74	Tnfrsf11a	3.753144925	1.49E-07
75	Ccr5	3.68279221	3.31E-07
76	Cd14	3.580844822	7.14E-07
77	Raet1	3.566477816	0.000146583
78	Il18	3.53497497	1.92E-06
79	Cfp	3.520792003	1.81E-05
80	C1qb	3.480995623	5.01E-08
81	Fcgr3	3.43212001	2.58E-06

82	Cxcl16	3.386042309	8.08E-08
83	Cd274	3.355668031	0.002218232
84	Tgfb2	3.273196014	5.66E-07
85	C5ar1	3.053364873	1.17E-06
86	Siglec1	3.025974857	3.98E-05
87	Csf3r	2.958775018	4.77E-05
88	Cd48	2.95733976	3.14E-07
89	Il4ra	2.921278694	1.62E-06
90	Bst1	2.881260294	0.000123616
91	Itgb2	2.843366363	8.42E-06
92	Tlr9	2.82666321	1.22E-05
93	Pou2f2	2.81551727	0.003235867
94	Irf5	2.756046062	1.12E-05
95	Csf2rb	2.728484787	0.000222699
96	Abcg1	2.726972214	1.47E-06
97	Socs1	2.649095768	0.000433652
98	Socs3	2.602142554	0.000212948
99	Nlrp3	2.590983809	0.001027969
100	Blnk	2.583451858	5.67E-05
101	Maf	2.360512299	7.51E-06
102	Isg20	2.351367437	0.000731659
103	Fos	2.346157718	0.005511573
104	Ddx58	2.326401331	5.72E-06
105	Alcam	2.323983781	1.28E-05
106	Thbs1	2.316264488	1.79E-05
107	Batf	2.256364275	0.00020071
108	Il2rg	2.234884075	0.000276662
109	Oas2	2.185403992	6.23E-05
110	Ly86	2.184041091	0.000353233
111	Tlr6	2.180864294	5.78E-05
112	Stat2	2.113450027	0.004435959
113	Ticam2	2.101180353	1.60E-05
114	Tlr2	2.076134629	0.000569834
115	Anxa1	2.059936692	0.000124898
116	Ptprc	2.04854559	0.000467039
117	Ddx60	2.015167835	0.003814171
118	Bid	2.006526208	0.000709943
119	Vwf	0.498926775	0.000320748
120	Stat5b	0.497786842	0.000869716
121	Map2k4	0.497338492	0.000197455
122	Cyfip2	0.497028333	0.001850584

123	Slamf1	0.496511831	0.008744683
124	Mapk1	0.495823996	0.000138179
125	Crebbp	0.49520576	0.001056651
126	Il1rl1	0.494794031	0.007283819
127	Pparg	0.493321476	0.001245811
128	Cd2	0.493047997	0.007821766
129	Reps1	0.492126121	0.000282161
130	Rps6	0.490899635	0.001804881
131	St6gal1	0.490729532	0.000374184
132	Cd36	0.489336906	0.000546828
133	Ceacam1	0.488963947	0.000589089
134	Gpi1	0.488151206	0.000236532
135	Tfrc	0.487576332	0.000387542
136	Il11ra1	0.486529774	0.001546294
137	Atf2	0.485552769	0.000175005
138	Il16	0.48494734	0.001345785
139	Egfr	0.482533162	0.000532461
140	Tnfrsf10b	0.482065136	0.000478417
141	Csf1	0.480131002	0.000318102
142	Myc	0.480064447	0.00049002
143	Hmgb1	0.479964631	0.000148873
144	Nfatc1	0.478735269	0.001712739
145	Mapkapk2	0.4765832	9.45E-05
146	Smad3	0.47355372	0.000573484
147	Erbb2	0.473258394	0.001396521
148	Cx3cr1	0.472537261	0.000582292
149	Mef2c	0.47243901	0.000147918
150	Cmah	0.471621044	0.001513633
151	Fadd	0.471065637	0.000779832
152	Cd34	0.4695335	5.20E-06
153	Prkce	0.468623108	0.000326222
154	Klrd1	0.465999399	0.007133788
155	Cspg4	0.46554741	9.21E-05
156	Ccnd3	0.461755164	0.000190975
157	Pla2g6	0.460636297	0.000477276
158	Icam2	0.459998161	1.96E-05
159	Dock9	0.458025555	0.000325815
160	Eng	0.457391037	5.48E-05
161	Smad4	0.456630775	0.000130502
162	Itga1	0.456282744	7.85E-05
163	Tmed1	0.4557454	0.000298273

164	Col1a1	0.455114041	2.88E-05
165	Sigirr	0.455082495	0.000283923
166	Igf1r	0.453602344	0.000899744
167	Il6st	0.453350883	2.60E-05
168	Cd81	0.452816992	7.25E-06
169	Hspb2	0.452754222	0.000252756
170	Nrp1	0.451187811	2.71E-05
171	Egr1	0.449782676	4.44E-05
172	Clu	0.449252987	0.000136026
173	Ccl19	0.448475165	0.001223559
174	Mcam	0.448350838	4.96E-06
175	Bcl2	0.448195479	0.000952225
176	Tab1	0.44813335	0.000297892
177	Irak1	0.44807123	0.000122792
178	Map3k5	0.447605603	0.000241269
179	Pecam1	0.444051835	7.66E-06
180	Cd200	0.441167983	7.56E-05
181	A2m	0.441015113	0.00038528
182	Thbd	0.433198454	3.87E-05
183	Gtf3c1	0.432658305	0.000264802
184	Ccl24	0.430862666	0.002324262
185	Vegfa	0.426642621	0.000277798
186	Cd276	0.426317446	0.000128221
187	Timd4	0.425785876	0.002207442
188	Cd163	0.421148228	3.97E-05
189	C1qbp	0.420477352	5.83E-05
190	Tgfb3	0.419342221	0.000101212
191	Angpt1	0.417775559	5.86E-05
192	Pdgfrb	0.415897524	3.24E-05
193	Rorc	0.411966805	0.000150025
194	Ncr1	0.410883129	0.007900112
195	Twist1	0.408356189	7.53E-05
196	Tek	0.408327885	1.02E-05
197	Cd1d1	0.408271283	0.007600486
198	Cxcl12	0.407366712	4.93E-05
199	Gzmm	0.404693056	0.001350022
200	Fyn	0.404272507	2.11E-05
201	Ccr7	0.403292925	0.000317519
202	Tie1	0.40114619	8.30E-05
203	Selplg	0.399702915	0.000151903
204	Dpp4	0.399647508	0.001623082

205	Cdh5	0.399066201	0.00019274
206	Jam3	0.397850964	8.28E-06
207	Nt5e	0.395842933	1.82E-05
208	Kdr	0.389231399	5.32E-05
209	Ecsit	0.388988659	2.56E-05
210	Masp1	0.387535398	0.000185398
211	Thy1	0.384218795	1.21E-05
212	Dll4	0.383394089	0.000123173
213	Ets1	0.381988206	1.78E-06
214	Vegfc	0.377513463	2.46E-05
215	Ccl21a	0.368618402	1.62E-06
216	Tnfrsf12a	0.367725212	0.000867541
217	Fn1	0.358091456	0.000266675
218	Zfp13	0.356284098	4.24E-06
219	Cd207	0.352305768	0.000393699
220	Cma1	0.346853897	0.000754336
221	Cd3d	0.340540903	0.000373767
222	C2	0.32894083	0.000224547
223	Rrad	0.325651325	5.19E-05
224	Lck	0.322976253	0.000326122
225	Smpd3	0.30120132	3.28E-05
226	Txnip	0.30038819	1.21E-05
227	Col4a1	0.296704765	3.05E-06
228	Msln	0.254952431	2.48E-05
229	Col3a1	0.244736299	4.63E-08
230	Gzma	0.239915787	4.37E-05

Supplementary Table 3. **Immune profiling gene panel in heart tissue from old and young mice exposed to *M.avium*.** Total RNA purified from sham or *M.avium* infected old and young mice hearts were used for NanoString analysis by using nCounter Mouse PanCancer Immune Profiling Panel. Total RNA (200ng) purified from the heart tissue and used for NanoString analysis. The relative expression of each transcript was normalized against the expression of the top Genes with counts > 100. Each value is the mean of biological triplicate and the ratios between means per target genes provides linear Fold change. Two-tailed Student's t test is used to select differentially expressed genes with values < 0.05.

Number	Gene	Fold change Control Old vs Control Young	p.Value
1	Ccl8	19.90280685	6.50E-06
2	Cxcl13	5.21837275	0.005280115
3	C6	4.143069438	0.001580204
4	Ccl5	4.038445304	5.77E-05
5	Zbp1	3.910627171	0.002226075
6	Fcgr4	3.820337246	0.002652368
7	Clec4n	3.620527728	1.80E-06
8	Clec7a	3.581589515	7.95E-06
9	Xcl1	3.444990489	0.002789936
10	Ccl12	3.385807615	7.36E-05
11	Ccl6	3.140294799	3.29E-05
12	C4b	2.986384653	0.000872979
13	H2-Aa	2.877069356	0.005589176
14	Ccr2	2.868506923	0.000119392
15	Cfb	2.866519315	0.008525782
16	Ifit1	2.819227691	0.003577843
17	Il1rn	2.800142018	0.006949722
18	Cxcr6	2.681796933	0.000811967
19	Psmb8	2.672889136	0.001889241
20	Trem2	2.553896395	0.000515278
21	Irf7	2.479759144	0.009601642
22	Casp1	2.456322517	4.93E-06
23	Psmb9	2.456322517	0.003804134
24	Ifit3	2.442739468	0.009916946
25	Fcgr3	2.424689481	7.13E-05
26	Ccl9	2.422841453	4.67E-05
27	Klra2	2.419485008	0.004431991
28	S100a8	2.416635687	0.006051627
29	Fcgr1	2.377260496	0.001518648
30	Ccr5	2.374460901	2.51E-05
31	Lgals3	2.364442405	0.000246007
32	Slamf7	2.341284089	0.000147252
33	Spp1	2.325756405	0.000209404
34	H2-DMb1	2.321086036	0.004914469
35	Msr1	2.276156886	7.62E-05
36	Ctss	2.271271231	0.0003514
37	Ly86	2.252145444	0.00025404
38	Isg15	2.24575415	0.009084413
39	Clec5a	2.210235352	0.000114217

40	Il23r	2.182830338	0.008629666
41	Il7	2.173319077	0.001251389
42	C1qb	2.167752427	9.26E-06
43	Cxcl11	2.157110378	0.009876862
44	Cd207	2.129773487	0.004366008
45	C1qa	2.10657605	1.09E-05
46	Lyz2	2.044290185	0.000215092
47	Herc6	2.033690306	0.001888239
48	Cd200r1	2.02791896	0.000156901
49	Cxcl16	2.008195884	3.17E-05
50	Klrk1	2	0.00180635
51	Ppbp	0.488794515	0.003430422
52	Masp1	0.481964904	0.001555206
53	Igf2r	0.476153948	0.000482866
54	Cspg4	0.44664484	5.63E-05
55	Crebbp	0.439306576	0.000293648
56	Itgb3	0.424136339	0.000898726
57	Igf1r	0.422962012	0.000454616
58	Nfatc2	0.40779048	0.000223952
59	Itga2b	0.393353957	0.000431738
60	Col4a1	0.380297405	3.12E-05
61	Gtf3c1	0.373479251	6.15E-05
62	Fn1	0.366859614	0.000327032
63	Sell	0.361608376	0.006282286
64	Col3a1	0.361007321	1.71E-06
65	Card11	0.338094863	0.001160324
66	Ncam1	0.326600748	1.12E-05
67	Col1a1	0.321034444	6.25E-07
68	Tfrc	0.299805759	2.71E-06
69	Pax5	0.241534303	0.003177184
70	Cd22	0.224393066	0.001909524
71	Cd19	0.132393115	4.57E-05
72	Dmbt1	0.064654742	1.07E-06

Supplementary Table 4. **Immune profiling gene panel in heart tissue from old and young mice.** Total RNA purified from hearts of old and young mice were used for NanoString analysis by using nCounter Mouse PanCancer Immune Profiling Panel. Total RNA (200ng) purified from the heart tissue and used for NanoString analysis. The relative expression of each transcript was normalized against the expression of the top Genes with counts > 100. Each value is the mean of biological triplicate and the ratios between means per target genes provides linear Fold

change. Two-tailed Student's t test is used to select differentially expressed genes with values < 0.05.
